# Juvenile xanthogranuloma mimicking malignancy at uncommon sites in children: five cases and a systematic review

**DOI:** 10.3389/fonc.2026.1976218

**Published:** 2026-10-23

**Authors:** Shan Zhang, Weijian Chen, Haomiao Zhou, Qiuhong Ma, Rong Wen, Li Du

**Affiliations:** 1 Department of Pathology, The Affiliated Children's Hospital of Xiangya School of Medicine, Central South University (Hunan Children's Hospital), Changsha, Hunan, China; 2 Department of Radiology, The Affiliated Children's Hospital of Xiangya School of Medicine, Central South University (Hunan Children's Hospital), Changsha, Hunan, China; 3 Department of Critical Care Medicine, Hunan Aerospace Hospital, Hunan Normal University, Changsha, Hunan, China

**Keywords:** diagnostic pitfall, extracutaneous, juvenile xanthogranuloma, malignancy mimic, pediatric, systematic review

## Abstract

**Introduction:**

Juvenile xanthogranuloma (JXG) typically presents in childhood as self-limited cutaneous lesions, but extracutaneous involvement is uncommon and may raise concern for malignancy. Although previous reviews have summarized the clinical spectrum of extracutaneous JXG, to our knowledge no study has systematically applied predefined criteria to quantify malignancy-mimicking presentations before histopathological diagnosis, or their diagnostic and management consequences, at the patient level.

**Methods:**

We retrospectively analyzed five pediatric patients with JXG at uncommon anatomical sites and conducted a PRISMA 2020-guided systematic review of PubMed/MEDLINE, Embase, and CNKI (January 2006–September 2026; no language restriction).

**Results:**

The five cases from our institution (three boys and two girls; age range, 4–12 years) involved the chest wall/pleural cavity, spinal epidural space, Meckel's cave, kidney, and pineal region. All lesions were mass-forming; three were initially radiologically suspicious for malignancy, whereas two showed other tumor-like growth patterns. Histopathology demonstrated histiocytic proliferations expressing CD68 and/or CD163; CD1a and langerin were negative in the cases tested. Targeted sequencing detected a PIK3CA p.H1047R variant in one case (variant allele frequency, 1.96%); given its low allele frequency and the absence of orthogonal validation, this finding was interpreted cautiously as supportive rather than diagnostic. After linking reports at the patient level, the systematic review included 150 reports corresponding to 147 unique patients. A malignancy-mimicking presentation before histopathological diagnosis was documented in 15 of 147 patients (10.2%; in sensitivity analysis, 17/147 [11.6%]). Diagnostic or management consequences were documented in 11 of 15 patients (73.3%), including enucleation for suspected retinoblastoma.

**Discussion:**

Major diagnostic challenges included unusual anatomical sites, tumor-like imaging findings, variable histopathologic features, and overlap with other histiocytic disorders. Diagnosis requires integration of clinical, radiological, pathological, immunophenotypic, and selective molecular data. Molecular findings are supportive rather than diagnostic, and no known molecular alteration is specific to JXG. We propose a practical diagnostic framework for distinguishing uncommon-site JXG from malignancy and other tumor-like processes. This framework is based on evidence from small, heterogeneous case series and institutional experience and is not intended to serve as an evidence-based management standard.

## Graphical Abstract

**Figure:**
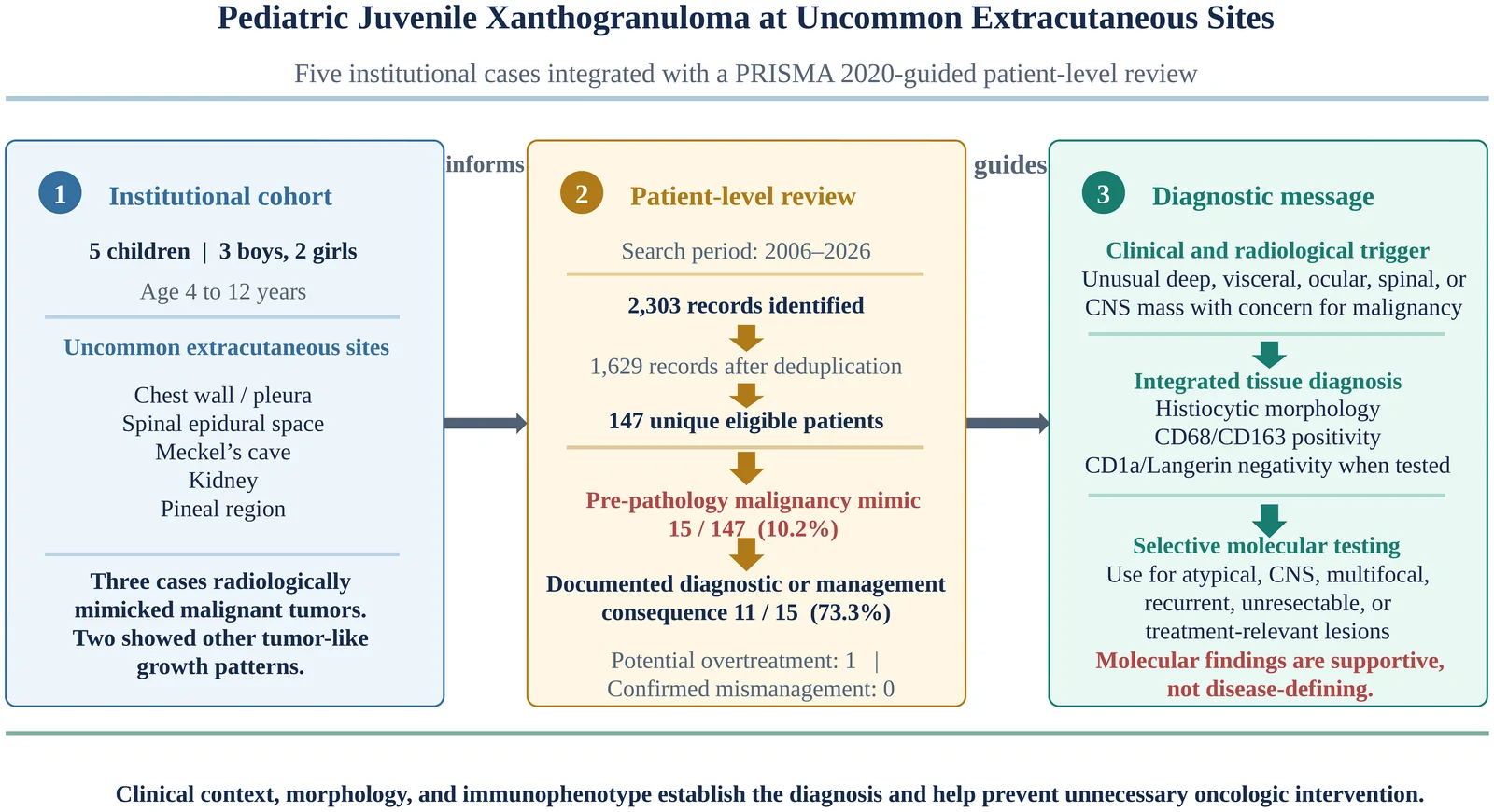
Based on five institutional cases and a PRISMA 2020-guided systematic review, this graphical abstract summarizes malignancy-mimicking presentations, diagnostic pitfalls, and a proposed practical diagnostic framework for pediatric extracutaneous juvenile xanthogranuloma. The framework is informed by heterogeneous case-level evidence and institutional experience and is not intended as an evidence-based management standard.

## 1 Introduction

Juvenile xanthogranuloma (JXG) is a common non-Langerhans cell histiocytic disorder of childhood. It typically presents as solitary or multiple yellow-red cutaneous papules or nodules and often regresses spontaneously (1, 2). In the current World Health Organization classification, JXG is included within the xanthogranuloma family of histiocytic and dendritic cell neoplasms (1, 2). Extracutaneous involvement is uncommon but may occur in the eye, central nervous system (CNS), bone, deep soft tissue, lung, liver, spleen, and other sites (3, 4). When it arises at deep, visceral, or anatomically unusual sites, JXG may present as a mass without characteristic skin lesions and may therefore be mistaken for a malignant or other neoplastic process, including sarcoma, lymphoma, germ cell tumor, Wilms tumor, or peripheral nerve sheath tumor. CNS involvement is of particular clinical concern because of the potential for neurological morbidity (3, 4). Diagnosis relies primarily on histopathological evaluation combined with immunophenotyping. Typical lesions show a histiocytic proliferation with foamy histiocytes and Touton giant cells, although these features may be variable, particularly in early or atypical lesions. Lesional cells commonly express CD68 and CD163 and are negative for CD1a and langerin when tested (5). Variable S100 and CD4 expression may also occur and should be interpreted in the context of morphology and clinical findings (5). Molecular alterations involving the MAPK pathway and kinase fusions have been reported in selected histiocytic neoplasms, including some JXG-family lesions, particularly in clinically aggressive or recurrent disease, CNS involvement, or anatomically atypical lesions (6, 7). Reported alterations include BRAF V600E, ALK rearrangements, and NTRK1 fusions (6–11). However, no known molecular alteration is specific to JXG; molecular findings should therefore be considered supportive rather than diagnostic. In selected unresectable or refractory cases, molecular testing may inform the use of targeted therapy (4, 8–11). Previous systematic reviews have summarized the clinical spectrum and treatment of extracutaneous JXG (12–14), but the frequency and consequences of malignancy-mimicking presentations before histopathological diagnosis have not been systematically quantified at the patient level. Here, we describe five pediatric cases of JXG at uncommon sites—the chest wall with rib destruction, spinal epidural space, Meckel’s cave, kidney, and pineal region—and a PRISMA 2020-guided systematic review that applied a predefined, patient-level definition of malignancy-mimicking presentation before histopathological diagnosis. We integrate imaging–pathology correlations that underlie diagnostic pitfalls and propose a practical diagnostic framework. This framework is based on heterogeneous case-level evidence and institutional experience and is not intended to serve as an evidence-based management standard.

## 2 Materials and methods

### 2.1 Clinical data

Five pediatric cases of juvenile xanthogranuloma (JXG) at uncommon anatomical sites were retrospectively identified from the pathology archives of Hunan Children’s Hospital between January 2017 and March 2026. Demographic characteristics, clinical presentation, imaging findings, surgical and medical treatment, and follow-up information were obtained from the medical records. All cases underwent pathological review by experienced pathologists. Diagnostically challenging cases were additionally reviewed by external experts when external consultation was obtained.

### 2.2 Histopathological examination

Surgical specimens were routinely processed, embedded in paraffin, sectioned at 4 μm, and stained with hematoxylin and eosin for histological evaluation. Predominant cellular morphology, inflammatory background, multinucleated giant cells, necrosis, calcification, and involvement of adjacent structures were assessed when present. The diagnosis of JXG was based on integration of the clinical context, imaging findings, morphology, and available immunophenotypic results. Histiocytic morphology, including foamy, epithelioid, spindle-cell, or multinucleated features, was interpreted in the context of the individual lesion. Touton-type giant cells were supportive when present but were not required, as they may be inconspicuous or absent in deep or morphologically atypical lesions. Expression of CD68 and/or CD163 supported histiocytic differentiation. CD1a and langerin negativity, when assessed, provided evidence against Langerhans cell histiocytosis. Markers that were not performed in a given case were not presumed to be negative and are listed as not available in **Table 1**.

**Table 1:** Clinicopathological features of five cases of juvenile xanthogranuloma arising in uncommon pediatric sites.

Case	Age/ Sex	Primary site	Presenting symptoms	Key imaging finding	Initial radiological diagnosis	Key IHC/molecular findings	Final diagnosis	Treatment	Outcome/ follow-up
Case 1	4 y 2 m/F	Chest wall/pleural cavity (ribs 8-10)	Chest wall mass (5 d) with fever (7 d); history of multiple colonic polyps (biopsied; juvenile polyps), chronic anemia, and growth retardation (unrelated)	Large heterogeneous mass with hemorrhage, bone erosion, and heterogeneous enhancement	Askin tumor/pleuropulmonary blastoma	CD163+, focal CD68+, focal S100+, focally positive, TFE3+, Ki-67 ~20%; CD1a-, langerin-, ALK-, LCA-, MPO-, CD30-, CD123-, SATB2-, myogenin-, SALL4-, CR-, INI1 retained, CD34 vessels only; focal CK+, focal desmin+, rare MyoD1+, focal weak CD99+, focal weak NKX2.2+; NGS: PIK3CA p.H1047R mutation (VAF 1.96%); no clinically significant variants in BRAF, NTRK1, NTRK2, NTRK3, or RET	JXG with locally aggressive behavior	Complete resection (tumor and rib resection, right lower-lung wedge resection, pleurolysis) + low-dose prednisone (1 mg/kg/d, 10 courses) for locally invasive disease	Good response; latest imaging showed pleural adhesions with improvement; no treatment-related toxicity; disease-free at 19 months
Case 2	5 y 5 m/F	Spinal canal, epidural (T5-T6)	Back discomfort and lower limb weakness	Well-defined spindle-shaped extradural mass compressing the spinal cord	Nerve sheath tumor	CD68+, CD163+, CD4+, scattered S100+, Ki-67 20%; CD1a-, langerin-, ALK-	Solitary spinal epidural JXG (single-system)	Complete resection of the epidural mass; no adjuvant therapy	Disease-free at 42 months
Case 3	12 y 5 m/M	Middle cranial fossa (Meckel’s cave)	Facial numbness and ocular discomfort	Lobulated dumbbell-shaped enhancing mass along the trigeminal nerve	Trigeminal neuroma/meningioma	CD68+, CD163+, CD4+, scattered S100+, Ki-67 5%; CD1a-, Langerin-, BRAF V600E-	Solitary JXG of Meckel’s cave (single-system)	Complete resection of the Meckel’s cave lesion; no adjuvant therapy	Disease-free at 39 months
Case 4	8 y 3 m/M	Right kidney	Gross hematuria	Large renal mass suggestive of Wilms tumor	Wilms tumor	CD68+, CD163++, CD4++, fascin+, vimentin++, BRAF V600E (VE1) partial weak+, TFE3 weak+, focal EMA+, focal PGP9.5+, Ki-67 ~10% (hotspot); CD1a-, langerin-, S100-, ALK-, WT1-, PAX8-, CK-, CK8/18-, CD10-, CD56-, cyclin D1-, desmin-, myogenin-, MyoD1-, HMB45-, p53-, TRK-, CD21-, CD35-, CD123-, CD30-, CD31-, CD34 vessels only; INI1, BRG1, H3K27me3, and Rb retained; NGS: no pathogenic genetic alterations detected	Juvenile xanthogranuloma-family lesion with atypical histologic features	Radical nephrectomy with retroperitoneal lymph-node dissection; no adjuvant therapy	Disease-free at 34 months
Case 5	5 y 9 m/M	Pineal region	Gait instability, weakness, and hand tremor	Enhancing pineal mass (2.7 × 2.4 × 2.3 cm); PET-CT: multifocal nodules in the ventricles and cisterns and bilateral frontoparietal calvarial lesions	Pineoblastoma/germinoma	CD68+, CD163+, S100 diffuse+, Ki-67 1%; CD1a-, lin28-, c-KIT-, BRAF V600E-, ALK-	Disseminated JXG (multifocal CNS and calvarial involvement)	Complete resection of the pineal mass + Group 2 multiagent chemotherapy (vincristine 1 mg, mercaptopurine 30 mg, prednisone 27.5 mg; 6 cycles) for multifocal CNS and calvarial disease	Disease-free at 50 months

For morphologically atypical lesions, differential diagnoses were considered according to the anatomical site and the available clinicopathological evidence. In the renal lesion, marked nuclear pleomorphism, bizarre nuclei, and multifocal necrosis initially raised concern for histiocytic sarcoma. However, histiocytic sarcoma was not favored after integrating the histiocytic immunophenotype; negativity for CD1a, langerin, ALK, and epithelial, myogenic, and renal-lineage markers; external expert consultation; and the subsequent clinical course. The case-specific differential diagnostic assessment is summarized in **Supplementary Table S3**.

### 2.3 Immunohistochemistry and molecular analysis

Immunohistochemical staining was performed on 4-μm formalin-fixed, paraffin-embedded tissue sections. Heat-induced epitope retrieval was performed in a microwave oven at high power for 30 min using a ready-to-use retrieval solution (Tongling, China; pH 9.0). All primary antibodies were ready-to-use, prediluted reagents and were applied without further dilution. Endogenous peroxidase activity was blocked with 3% hydrogen peroxide for 5 min. Primary antibodies were incubated at room temperature for 30 min, followed by incubation with a ready-to-use polymer-based detection reagent (DD13, HRP-labeled anti-mouse/rabbit secondary antibody polymer; Xiamen Talent Biomedical Technology Co., Ltd., Xiamen, China) for 20–30 min at room temperature, according to the manufacturer’s protocol. Diaminobenzidine was used as the chromogen for 2–5 min. Slides were then rinsed in water, counterstained with hematoxylin, differentiated with 0.1% hydrochloric acid alcohol, blued, dehydrated, cleared, and mounted. The immunohistochemical panel was selected according to the clinical and pathological differential diagnosis and included CD68, CD163, S100, CD1a, langerin, ALK, BRAF V600E, and site-specific lineage markers where indicated. Staining was performed under the laboratory’s established quality-control procedures; external control tissues and/or internal positive controls were reviewed when available for the relevant diagnostic staining batches. Detailed information on antibody clones, suppliers, antigen-retrieval conditions, and detection procedures is provided in **Supplementary Table S2**.

Targeted next-generation sequencing (NGS) was performed in Cases 1 and 4 using DNA extracted from formalin-fixed, paraffin-embedded tumor tissue. For each tested case, fifteen unstained 10-μm-thick tissue sections were submitted for DNA extraction and sequencing. Molecular testing for Case 1 was performed in the Department of Medical Genetics, Hunan Children’s Hospital, whereas testing for Case 4 was performed at Beijing Children’s Hospital. Molecular results were interpreted in conjunction with the clinical, histopathological, and immunophenotypic findings and were regarded as supportive rather than diagnostic.

### 2.4 Literature search and data extraction

This systematic review was conducted using a PRISMA 2020-guided approach. PubMed/MEDLINE, Embase, and the China National Knowledge Infrastructure (CNKI) were searched from 1 January 2006 to 12 September 2026, without language restriction. The primary search strategy combined terms for JXG and its synonyms. An additional strategy incorporating imaging-related terms was used to assess search sensitivity. Complete search strategies are provided in **Supplementary Table S1**. The search period was selected to reflect contemporary diagnostic practice, including routine cross-sectional imaging, standardized immunohistochemical assessment, and molecular testing, in accordance with the study objective of characterizing malignancy-mimicking presentations before histopathological diagnosis. A total of 2,303 records were identified (PubMed/MEDLINE, 794; Embase, 1,402; CNKI, 107). After removal of 674 duplicate records (662 between PubMed/MEDLINE and Embase and 12 within Embase), 1,629 records underwent title and abstract screening. Of these, 1,329 records were excluded and 300 full-text reports were assessed for eligibility. After full-text assessment, 150 reports were excluded and 150 reports were included in the systematic review (**Figure 1**). Patients aged ≤18 years with a definitive diagnosis of extracutaneous or extracutaneous-first JXG and sufficient extractable patient-level data were eligible. Adult cases; skin-only or skin-first JXG; uncertain diagnoses; non-JXG xanthogranulomatous disorders; animal studies; unrelated records; and reports without extractable patient-level data were excluded. Two authors independently screened the records. As a verification aid, a large language model (Claude, Anthropic) independently re-adjudicated the full screening pool (n = 1,390) and the additional Embase-only records (n = 209), and the corresponding author adjudicated disagreements. All AI-assisted outputs were reviewed and verified by the authors, who take full responsibility for the accuracy and integrity of the work.

**Figure 1:**
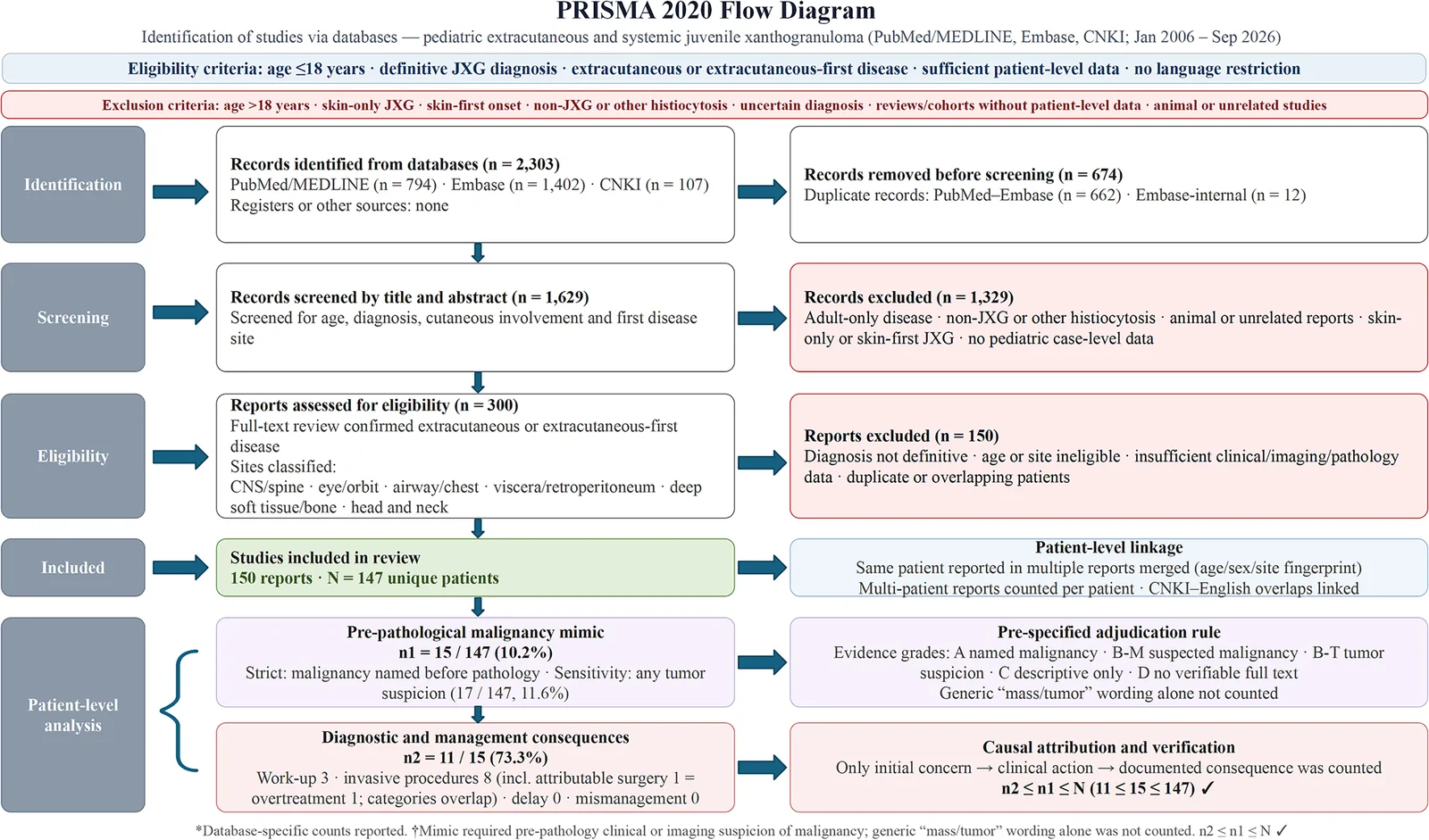
PRISMA 2020 flow diagram for the systematic review. PubMed/MEDLINE, Embase, and CNKI were searched without language restriction for reports of pediatric extracutaneous or systemic juvenile xanthogranuloma published between 1 January 2006 and 12 September 2026. After removal of 674 duplicates, 1,629 records underwent title and abstract screening (1,329 excluded), and 300 reports were assessed in full text (150 excluded). A total of 150 reports were included, corresponding to 147 unique patients after patient-level linkage. A pre-pathology malignancy-mimicking presentation was identified in 15 of 147 patients (10.2%; sensitivity analysis, 17/147 [11.6%]), with diagnostic or management consequences in 11 of 15 patients (73.3%).

For each eligible patient, age, sex, anatomical site, clinical and radiological presentation, pre-pathology diagnostic impression, pathological and molecular findings, treatment, and outcome were extracted. Multi-patient reports were expanded into individual patient records before patient-level linkage. Reports describing potentially overlapping patients were assessed using a predefined patient fingerprint comprising first and corresponding author, institution, publication year, age, sex, exact anatomical site, lesion multiplicity, clinical presentation, imaging features, treatment, follow-up, and molecular findings. Records were classified as confirmed duplicates, probable duplicates, possible overlaps, independent patients, or insufficiently documented (**Supplementary Table S4**). When a conference abstract and a full report described the same patient, the full report was retained and the conference abstract was not counted independently. Following patient-level linkage, the 150 included reports corresponded to 147 unique eligible patients. A pre-pathology malignancy-mimicking presentation required documented clinical or imaging suspicion of a named malignancy before histopathological diagnosis (grade A) or explicit wording that malignancy was suspected (grade B-M). Generic tumor suspicion (grade B-T), descriptive differential diagnoses without explicit malignant concern (grade C), and cases without verifiable full text (grade D) were not included in the primary analysis. Diagnostic or management consequences were counted only when the sequence of initial malignant concern, clinical action, and subsequent consequence was explicitly documented. Proportions are reported with explicit numerators and denominators. Conference abstracts were eligible when sufficient patient-level data permitted confirmation of age, extracutaneous JXG diagnosis, and the relevant study variables; appraisal of abstract-only reports was limited to the domains verifiable from that source.

### 2.5 Quality assessment

Study quality was appraised using the JBI critical appraisal checklists for case reports (eight items) and case series (ten items). Appraisal was performed at the level of the primary report, with multiple patients from the same report sharing a single report-level appraisal. Critical appraisal was based on the fullest accessible source for each report, and domains that could not be verified from the available source were rated as unclear. No numerical quality score was calculated, and no missing information was imputed as reported. Per-study ratings are provided in **Supplementary Table S5**.

## 3 Results

### 3.1 Clinical features

The cohort included three boys and two girls aged 4–12 years. Lesions involved the chest wall/pleural cavity, spinal epidural space, Meckel’s cave, kidney, and pineal region. Symptoms varied according to lesion location and included a chest wall mass with fever, back pain with lower-limb weakness, facial numbness with ocular discomfort, hematuria, and gait instability with hand tremor. All five patients had mass-forming lesions on imaging. Initial imaging raised concern for malignancy in three cases; the suspected diagnoses were Askin tumor or pleuropulmonary blastoma, Wilms tumor, and pineoblastoma or germinoma, respectively. The remaining two lesions showed other tumor-like radiological growth patterns, with nerve sheath tumor, trigeminal schwannoma, or meningioma considered radiologically (**Figure 2**). Several lesions were associated with bone destruction or spinal cord compression. Cases 1–4 were single-system and Case 5 was multisystem (multifocal CNS and calvarial involvement).

**Figure 2:**
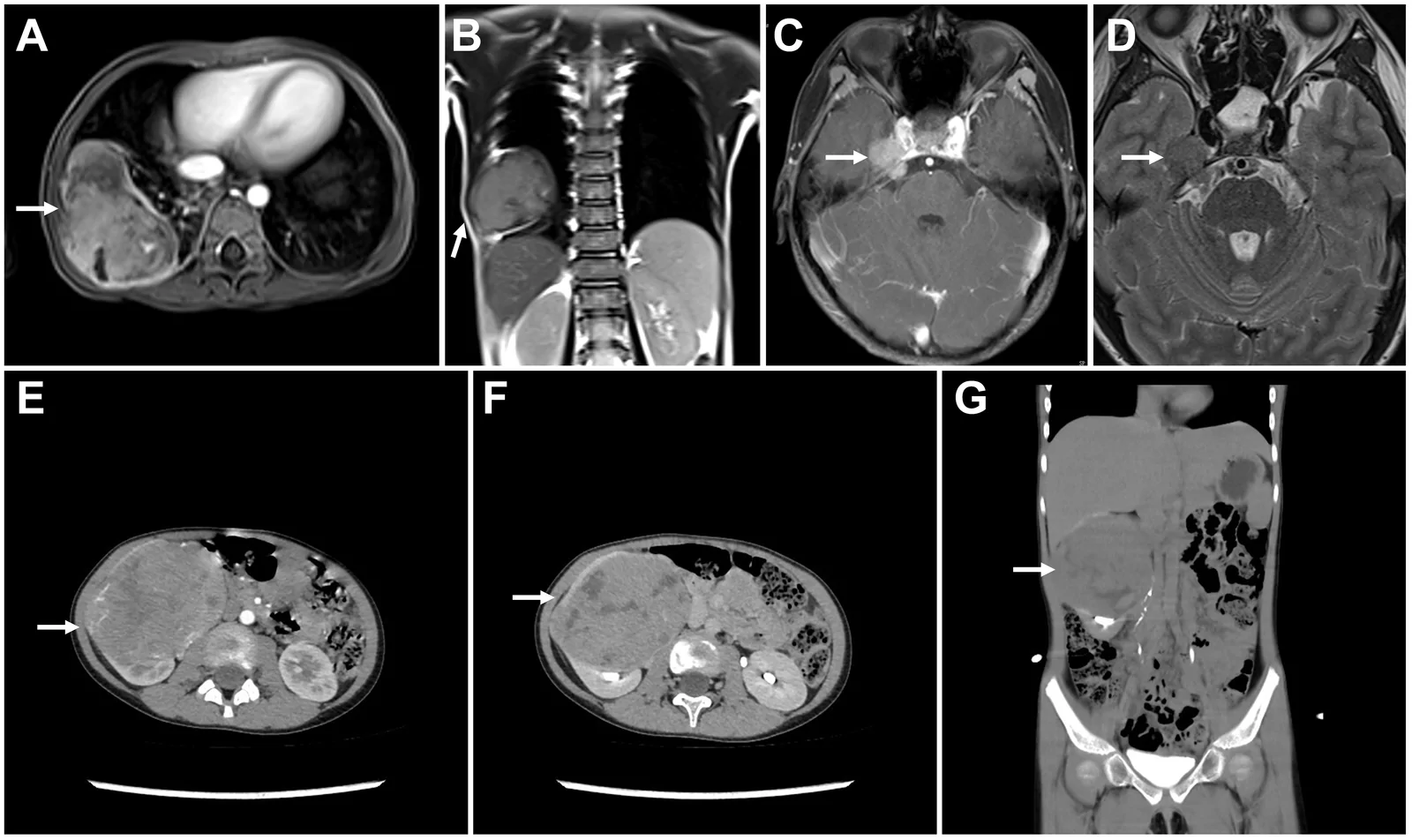
Representative radiological findings of Cases 1, 3, and 4. **(A, B)** Magnetic resonance imaging (MRI) of Case 1 demonstrating a heterogeneous chest wall and pleural mass with rib destruction and heterogeneous enhancement. **(C, D)** MRI of Case 3 showing a lobulated enhancing lesion involving Meckel’s cave. **(E–G)** Contrast-enhanced computed tomography (CT) of Case 4 demonstrating a large heterogeneous right renal mass with heterogeneous enhancement, occupying most of the kidney and associated with hydronephrosis.

No cutaneous lesions were documented in the five patients. Additional imaging included MRI of the involved region in Cases 1–3 and 5 and CT in Case 4. In Case 5, PET-CT showed additional multifocal nodules in the ventricles and cisterns, together with bilateral frontoparietal calvarial lesions. Bone marrow examination was performed in Cases 1 and 5 and showed hypercellular marrow with normal morphology; it was not performed in Cases 2–4.

### 3.2 Histopathological features

All lesions showed a histiocytic proliferation with abundant eosinophilic cytoplasm. Foamy histiocytes and Touton-type giant cells were present in several cases, accompanied by variable lymphocytic and eosinophilic inflammatory infiltrates. Deep lesions tended to be more cellular than conventional cutaneous JXG. Mitotic activity was generally low, although focal cytological atypia was present in some lesions. The renal lesion (Case 4) comprised diffuse sheets of spindle, short spindle, and round cells with enlarged eccentric nuclei, marked nuclear pleomorphism, multinucleated and bizarre giant cells, abundant eosinophilic cytoplasm, poor cohesion, and multifocal necrosis. The lesion involved the renal parenchyma, renal sinus, and perirenal adipose tissue; the ureteral margin was uninvolved. Representative histopathological findings are shown in **Figure 3**. Additional microscopic features are highlighted in **Figure 4**, including abundant foamy histiocytes in Case 3, focal necrosis with dystrophic calcification in Case 1, focal involvement of adjacent ganglion cells in Case 2, and a multinucleated giant cell with degenerative nuclear changes in Case 4.

**Figure 3:**
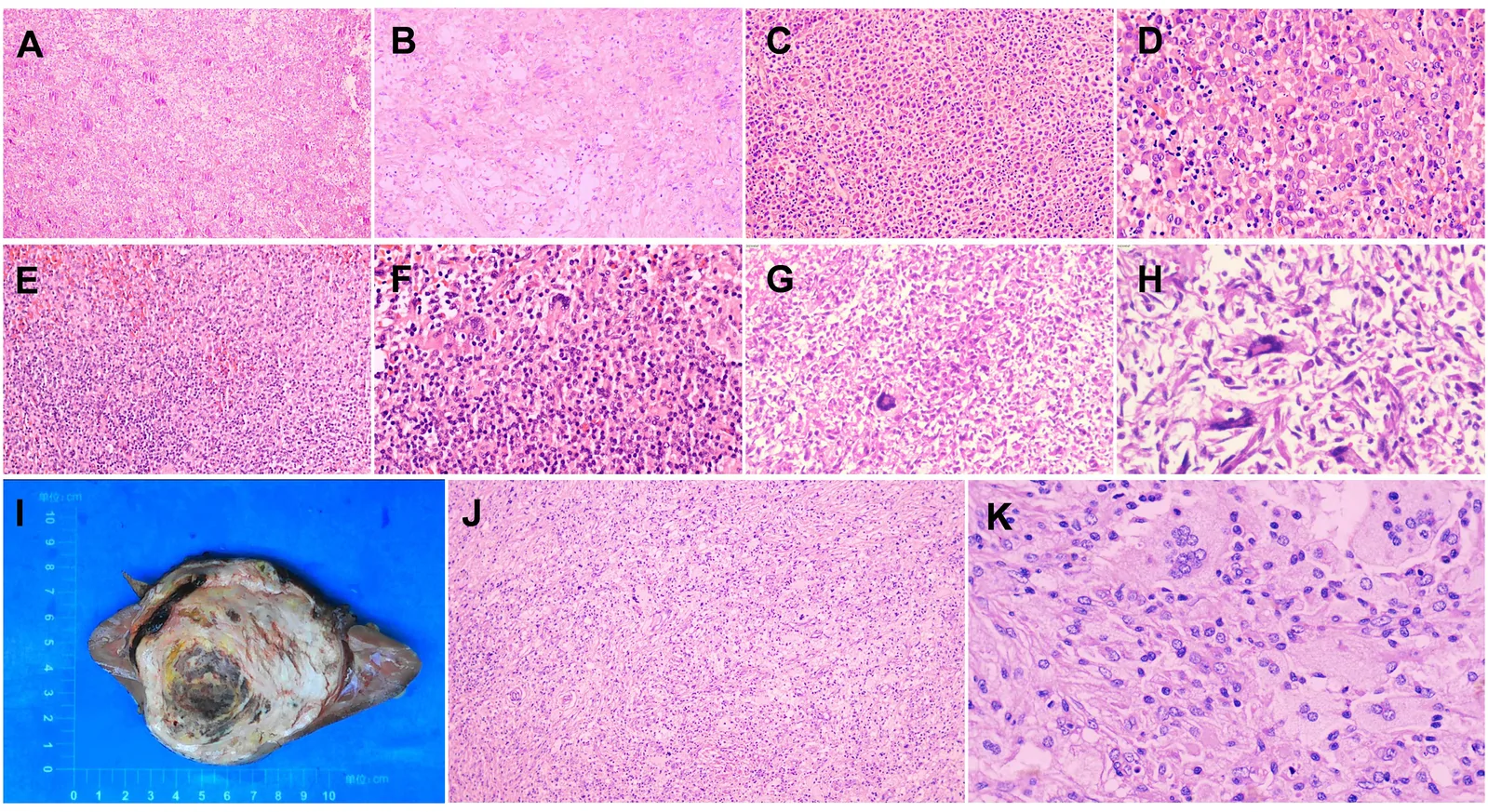
Representative histopathological findings of five cases of juvenile xanthogranuloma (H&E). **(A, B)** Case 1 showing histiocytic proliferation composed of foamy histiocytes, mononuclear histiocytes, and multinucleated giant cells [**(A)**, ×40; **(B)**, ×200]. **(C, D)** Case 2 showing a cellular histiocytic proliferation admixed with inflammatory cells [**(C)**, ×200; **(D)**, ×400]. **(E, F)** Case 3 showing mononuclear histiocytes with abundant eosinophilic cytoplasm and scattered inflammatory cells [**(E)**, ×200; **(F)**, ×400]. **(G, H)** Case 4 showing diffuse sheets of spindle, short spindle, and round histiocytic cells with enlarged eccentric nuclei, marked nuclear pleomorphism, multinucleated and bizarre giant cells, and abundant eosinophilic cytoplasm [**(G)**, ×200; **(H)**, ×400]. **(I)** Gross appearance of the renal mass in Case 4. **(J, K)** Case 5 showing foamy histiocytes within a fibro-inflammatory background, consistent with juvenile xanthogranuloma (J, ×100; K, ×400).

**Figure 4:**
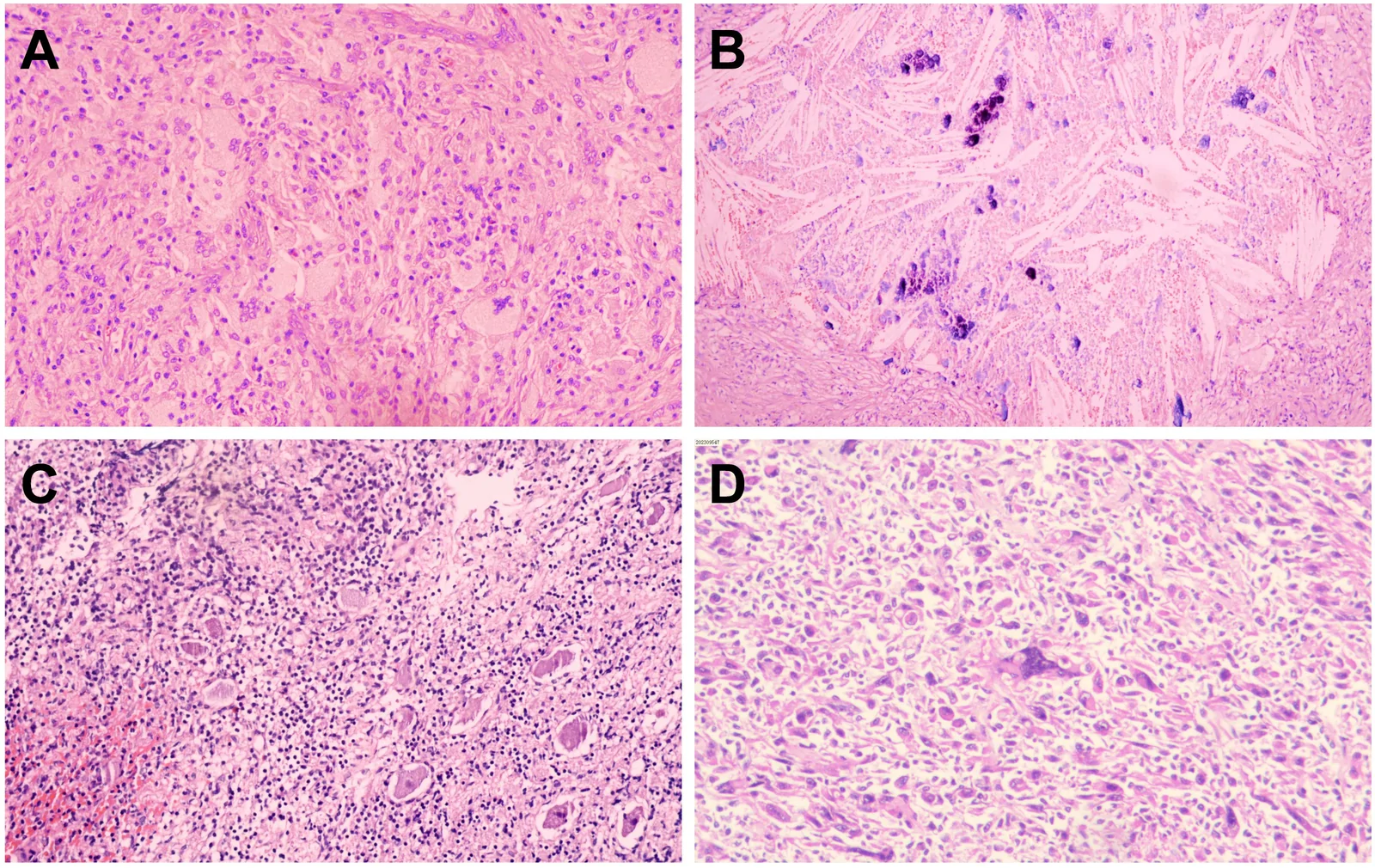
Characteristic histopathological features of juvenile xanthogranuloma (H&E). **(A)** Case 3 showing abundant foamy histiocytes (×400). **(B)** Case 1 showing focal necrosis with dystrophic calcification (×400). **(C)** Case 2 demonstrating focal involvement of adjacent ganglion cells (×200). **(D)** Case 4 showing a multinucleated giant cell with degenerative nuclear changes (×200).

At initial evaluation, these features raised genuine concern for histiocytic sarcoma. However, histiocytic sarcoma was not favored after integrating the histiocytic immunophenotype, external expert consultation at Beijing Children’s Hospital, and the subsequent clinical course. Immunohistochemistry showed positivity for CD163, CD4, and fascin, with negativity for CD1a, langerin, S100, ALK, and site-specific epithelial, myogenic, and renal-lineage markers; INI-1 expression was retained. The Ki-67 labeling index in hotspot areas was approximately 10%. Expert consultation led to a diagnosis of JXG, although the marked nuclear pleomorphism and multifocal necrosis were recognized as atypical histologic features. After surgical resection without adjuvant therapy, the patient remained free of disease for approximately 34 months. BRAF V600E immunoreactivity was weakly and focally positive and was considered a screening finding only, because the molecular report did not document a confirmatory BRAF alteration. TFE3 staining was positive; because staining intensity and extent were not quantified and TFE3 rearrangement testing was not performed, this finding was not interpreted as evidence of an Xp11 translocation-associated neoplasm. Although myeloid sarcoma could not be entirely excluded immunophenotypically because MPO staining and flow cytometry were not performed, it was not favored on overall clinicopathological assessment, particularly given the prolonged disease-free course without adjuvant therapy. The case-specific differential diagnostic considerations and available supporting or limiting evidence are detailed in **Supplementary Table S3**.

### 3.3 Immunohistochemical and molecular findings

Lesional histiocytes consistently expressed CD68 and CD163. CD1a and langerin were negative in the cases tested. S100 expression was focal in several lesions and diffuse in Case 5. The Ki-67 labeling index ranged from 1% to 20%. Focal BRAF V600E (VE1) immunoreactivity was observed in two cases (Cases 1 and 4), whereas ALK was negative in the cases tested. Because the immunoreactive cases were not confirmed as BRAF-mutant by sequencing, the staining results were treated as screening observations rather than molecular confirmation.

Targeted sequencing was performed in Cases 1 and 4. Case 1 harbored a PIK3CA p.H1047R mutation (variant allele frequency, 1.96%; tumor-cell content, 70%; mean effective depth, 2,668×; coverage, 99.88%; Q30, 95.72%); variant-supporting reads are shown in **Figure 5**, and the assay sensitivity threshold was not available from the sequencing report. No ALK alteration was identified; because the targeted panel assessed DNA-level alterations only, ALK rearrangements were not formally evaluated. No pathogenic alteration was identified in Case 4. No BRAF variant was molecularly confirmed in either immunoreactive case. Representative immunohistochemical and molecular findings are shown in **Figures 5**, **6**.

**Figure 5:**
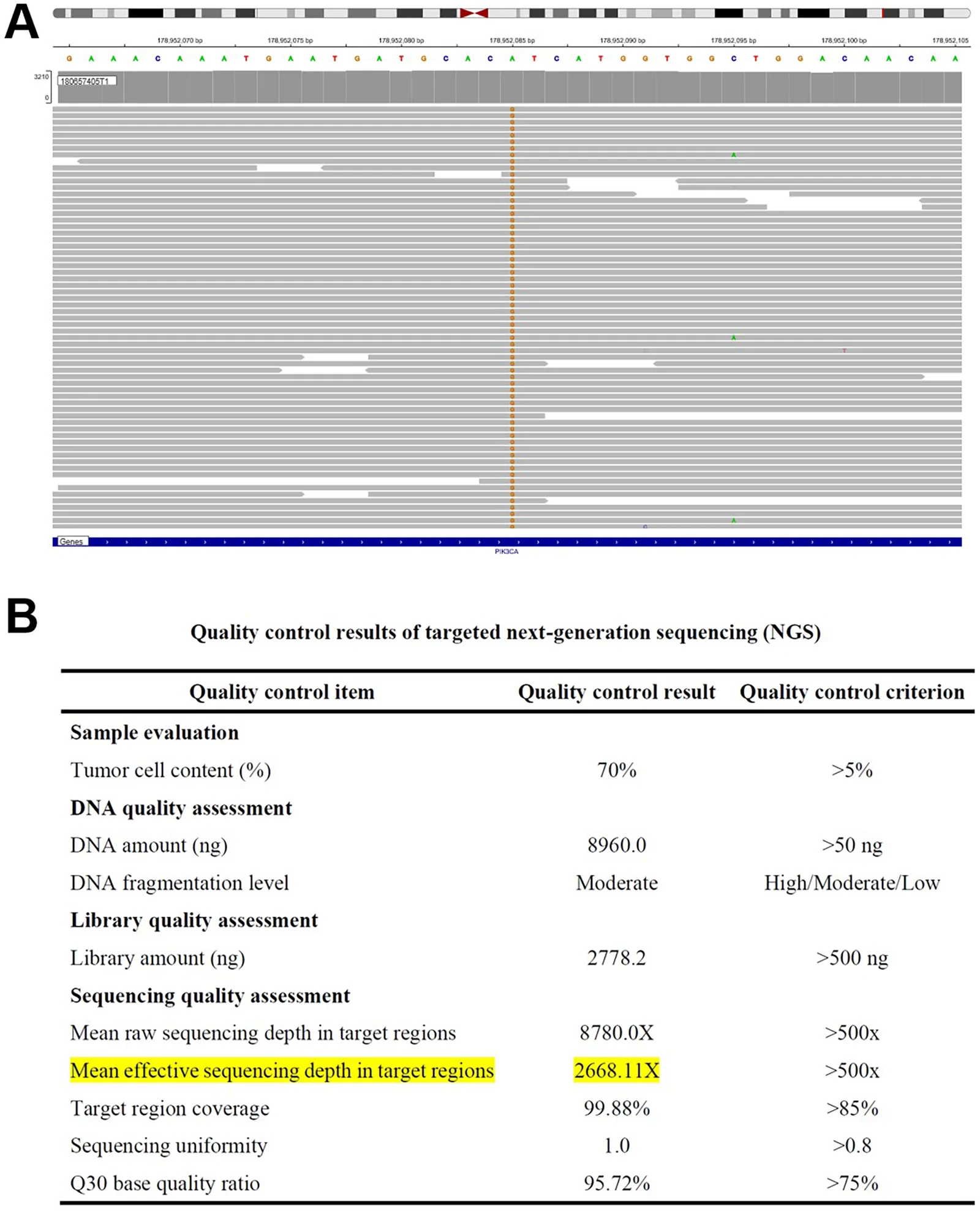
Molecular findings in Case 1. **(A)** Integrative Genomics Viewer image showing the PIK3CA p.H1047R mutation identified by targeted next-generation sequencing. **(B)** Quality control metrics showing adequate sequencing depth and variant-supporting reads at the PIK3CA p.H1047R locus, corresponding to a variant allele frequency of 1.96%.

**Figure 6:**
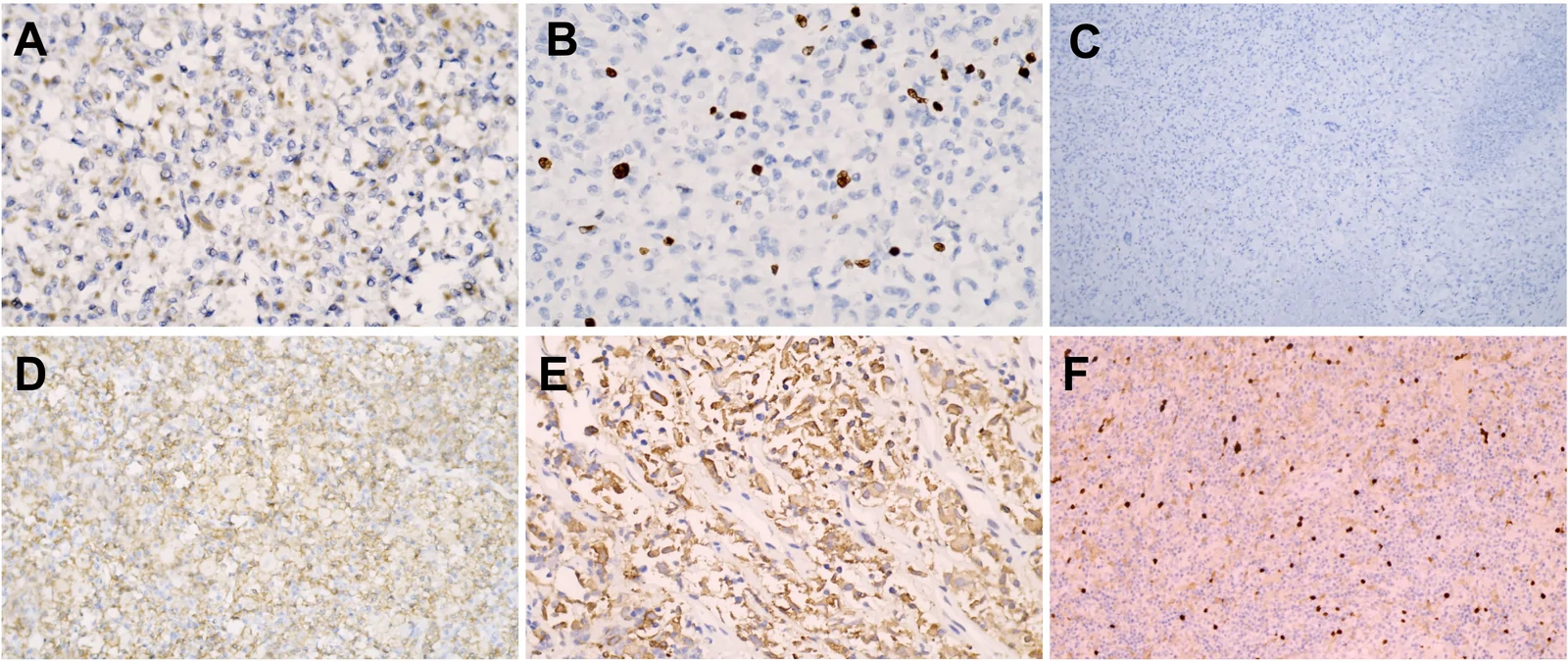
Immunohistochemical findings of juvenile xanthogranuloma. **(A)** Case 4 showing focal cytoplasmic immunoreactivity for BRAF V600E (×40). **(B)** Case 4 showing a Ki-67 labeling index of approximately 10% (×400). **(C)** Case 4 showing absence of Langerin (CD207) expression in the lesional cells (×100). **(D)** Case 4 demonstrating diffuse CD4 expression in the lesional histiocytes (×200). **(E)** Case 2 showing diffuse CD163 expression in the lesional histiocytes (×400). **(F)** Case 3 showing focal S100 expression in the lesional histiocytes (×200).

### 3.4 Treatment and follow-up

All patients underwent complete surgical resection. Adjuvant therapy was given to Cases 1 and 5: low-dose prednisone (1 mg/kg/d, 10 courses) in Case 1 for locally invasive disease with rib and muscle destruction, and Group 2 multiagent chemotherapy (vincristine, mercaptopurine, and prednisone; 6 cycles) in Case 5 for multifocal CNS and calvarial involvement; the remaining three patients received no adjuvant therapy. During follow-up (median, 39 months; range, 19–50), all patients were alive without recurrence or progression. **Table 1** summarizes the clinical, pathological, immunophenotypic, molecular, treatment, and follow-up data. Overall, the five cases were consistent with JXG despite their unusual sites and malignancy-mimicking presentation.

### 3.5 Systematic review findings

The search identified 2,303 records. After removal of 674 duplicates, 1,629 records underwent title and abstract screening, of which 1,329 were excluded. Full texts of 300 reports were assessed for eligibility; 150 were excluded, and 150 were included in the review (**Figure 1**). After patient-level linkage, the 150 included reports corresponded to 147 unique patients (**Supplementary Table S4**). Each retained report provided extractable patient-level data; cohort or review articles without such data were excluded during screening. Denominators vary by variable according to data availability. Reported sites included the CNS, eye or orbit, liver, lung, bone, kidney, and deep soft tissue; less common sites included the airway, pancreas, parotid gland, and temporal bone (12–17); individual-level clinicopathological data for all included patients are summarized in **Table 2**. A pre-pathology malignancy-mimicking presentation was documented in 15 of 147 patients (10.2%). In the sensitivity analysis, which additionally included cases with generic tumor suspicion, 17 of 147 patients met the definition (11.6%). Suspected malignancies included retinoblastoma, rhabdomyosarcoma, lymphoma, germ cell tumors, neuroblastoma, and sarcomas. Diagnostic or management consequences attributable to the initial malignant concern were documented in 11 of 15 patients (73.3%). These included additional diagnostic work-up in three patients and invasive diagnostic procedures in eight patients; categories were not mutually exclusive. One major surgery, enucleation for suspected retinoblastoma, was attributable to the initial malignant concern and constituted the single case of potential overtreatment. No documented diagnostic delay or confirmed mismanagement was identified.

**Table 2:** Individual-level clinicopathological data from published pediatric extracutaneous/systemic juvenile xanthogranuloma cases.

No.	Source	Age/sex	Primary site	Imaging or initial diagnosis considered	Key pathology/IHC	Molecular alteration/relevance	Initial diagnosis	Treatment
1	Kahn et al., 2020 (39)	Young child/NR	Subdural and extra-axial CNS spaces	Extra-axial process in shunt-treated hydrocephalus; chronic subdural hematoma or overshunting-related collection considered	Pathology-proven JXG	NR	Chronic subdural hematoma/overshunting-related collection	Diagnostic biopsy/surgical sampling
2	Matsunaga et al., 2020 (22)	16y/F	Iris and anterior chamber	Diffuse flake-like epi-iridic thickening; diffuse iris melanoma considered; anterior-segment OCT showed a stalagmite-like surface	FNAB: atypical histiocytic proliferation consistent with JXG	NR	Diffuse iris melanoma	Fine-needle aspiration biopsy followed by aggressive topical corticosteroids
3	Sosnowska et al., 2020 (18)	13 y/F	Endobronchial lesion, left main bronchus	Chest radiograph showed near-complete left upper-lobe atelectasis; CT showed a bronchial soft-tissue lesion and bronchoscopy showed a mass filling the left main bronchus	JXG after histological evaluation	NR	NR (atelectasis; bronchial mass)	Initial resection followed by further surgical treatment for recurrence
4	Al-Antary et al., 2022 (19)	13 mo/F	Pancreas	Pancreatic mass with elevated CA19-9; pancreatic neoplasm considered	JXG diagnosis	NR	Pancreatic neoplasm	Watch and wait
5	Rios et al., 2023 (40)	15 y/NR	Sellar, suprasellar, and multifocal CNS	Sellar/suprasellar lesion; craniopharyngioma, germ cell tumour, or pituitary lesion considered	Pathology confirmed JXG	NR	Craniopharyngioma/germ cell tumor/pituitary lesion	Partial resection
6	Maejima et al., 2024 (41)	5 mo/F	Deep intramuscular thigh	Mass invading the tensor fasciae latae; myeloid sarcoma or deep soft-tissue tumour considered	CD68/CD163+, CD1a/S100/MPO/CD34−	NR	Myeloid sarcoma/deep soft-tissue tumor	Needle biopsy followed by marginal resection
7	Suraneni et al., 2024 (25)	4 mo/M	Corneoscleral and anterior segment	Progressive ocular infiltration; uveitis, ocular malignancy, or vascular lesion considered	CD68+ histiocytes; malignancy excluded	NR	Uveitis/ocular malignancy/vascular lesion	Glaucoma drainage implant, lensectomy/vitrectomy, intravitreal bevacizumab, and methotrexate
8	Areal-Hidalgo et al., 2018 (42)	2 y/F	Solitary intra-axial CNS lesion at the floor of the third ventricle	Enhancing midline lesion; germinoma considered	Biopsy confirmed JXG; serum/CSF β-HCG and AFP were negative	NR	Germinoma	Vinblastine plus prednisone; cladribine
9	Sagga et al., 2023 (43)	3 y/M	T11-L1 extramedullary, intra- and extradural spinal mass	Spinal mass; meningioma, ependymoma, lymphoma, and neuroblastoma considered	Foamy histiocytes and Touton giant cells; CD68 and factor XIIIa strongly positive; Ki-67 up to 5%	NR	Meningioma/ependymoma/	Complete surgical resection
10	Irmola et al., 2018 (44)	5 y/F	T11 vertebral body and paravertebral region	Langerhans cell histiocytosis or Rosai-Dorfman disease considered	CD163+; CD1a−, S100−; low MIB-1	NR	Langerhans cell histiocytosis/Rosai-Dorfman disease	Neoadjuvant denosumab followed by surgical resection
11	Lalitha et al., 2011 (45)	Child/NR	Cerebellum, brainstem, thalami, and bilateral cerebral hemispheres	MRI showed multiple enhancing solid intracranial lesions	Prior skin biopsy and brain lesion biopsy confirmed JXG	NR	NR (multiple enhancing intracranial lesions)	Treatment not reported
12	Matsubara et al., 2016 (46)	2 y/F	Multifocal cerebral lesions	MRI showed multiple well-marginated lesions with reduced diffusion and diffuse enhancement; MR spectroscopy showed elevated taurine, choline, and glutamate	Brain biopsy confirmed JXG	NR	NR (multifocal cerebral lesions)	Brain biopsy; further treatment not reported
13	Gressot et al., 2013 (47)	6 wk/M	Optic apparatus, basal ganglia, lateral ventricles, and brainstem	Neuroimaging showed disseminated intracranial disease	CD68+, S100+, fascin+, factor XIIIa subset+; CD1a−, langerin−; no Birbeck granules	NR	NR (disseminated intracranial disease)	Open biopsy followed by postoperative chemotherapy
14	Cornips et al., 2009 (48)	2 mo/M	Temporal muscle, temporal bone, and dura	Not reported	Factor XIIIa+, CD68+; S100−, CD1a−, NSE−, EMA−	NR	NR (temporal muscle/bone mass)	Surgical excision with reconstructive skull procedure
15	De Paula et al., 2010 (49)	18 mo/F	Occipital bone and left extradural skull base	CT/MRI showed a contrast-enhancing extradural skull-base tumour partly occupying the posterior fossa, with secondary hydrocephalus	Histopathology and immunohistochemistry confirmed JXG	NR	NR (extradural skull-base tumor)	Partial surgical resection, chemotherapy, and conformal radiotherapy
16	Su et al., 2019 (50)	Child/F (exact age not reported)	CNS	Intracranial lesion with intense enhancement and high perfusion on multimodal MRI	Biopsy confirmed JXG (multimodal MRI case)	NR	NR (intracranial lesion)	Treatment not reported
17	Kumar et al., 2023 (51)	Not reported	Solitary intracranial lesion	Not reported	Brain biopsy confirmed JXG	NR	NR	Treatment not reported
18	Yaguchi et al., 2024 (52)	Not reported	Congenital intracranial lesion	Not reported	Biopsy confirmed JXG (letter)	NR	NR	Treatment not reported
19	Yang et al., 2021 (53)	13 mo/F	Posterior fossa, extra-axial	MR differential included PNET, atypical teratoid/rhabdoid tumour, haemangiopericytoma, paraganglioma, and glioblastoma	CD68 strongly+, CD163+, factor XIIIa+; Ki-67 10–20%; INI-1−, ALK−, langerin−	46, XX,ins(8;12)(q24.3;q24.3q15),del(12)(q13)	PNET/AT/RT/glioblastoma	Treatment not reported
20	Ferguson et al., 2015 (54)	11 y/F	Sellar region with disseminated intracranial disease	Sellar lesion initially diagnosed as granulomatous hypophysitis	Biopsy confirmed JXG	NR	Granulomatous hypophysitis (initial)	Subtotal resection followed by whole-brain radiotherapy and systemic chemotherapy
21	Calderon et al., 2021 (55)	9 y/M	Bilateral lateral ventricles, straight sinus, and right cerebellum	MRI showed extensive intracranial masses with gadolinium enhancement	IHC confirmed JXG	NR	Intraventricular tumors/metastatic disease	Treatment not reported
22	Nakasu et al., 2007 (56)	2 y/M	Left Meckel’s cave	Imaging showed a mass in the left Meckel’s cave in a child with left abducens nerve palsy	IHC confirmed JXG after partial resection	NR	NR (Meckel cave mass; abducens palsy)	Partial resection followed by stereotactic radiosurgery
23	Meyer et al., 2018 (57)	3 y/NR	Intraocular, left eye	B-scan showed complete vitreous opacity with punctate calcifications; infiltrative retinoblastoma was considered	CD68+, CD1a−, S100− (skin biopsy)	FGFR3 p.F386L missense (NGS)	Infiltrative retinoblastoma	Diagnostic enucleation
24	Kwok and Clark, 2015 (58)	19 mo/M	Bilateral intraocular (vitreoretinal) lesions	Endophytic retinoblastoma considered	Biopsy confirmed JXG	NR	Endophytic retinoblastoma	Examination under anaesthesia; treatment not reported
25	Fontanilla et al., 2009 (59)	Not reported	Bilateral uveal lesions	Uveal melanoma considered	Biopsy confirmed JXG	NR	Uveal melanoma	Systemic corticosteroids; further treatment not reported
26	Snijders et al., 2007 (60)	4 mo/NR	Pulmonary lesion	Cough, haemoptysis, and anemia; imaging findings not reported in the accessible record	Inflammatory cells, fibroblasts, and rare Touton giant cells	NR	NR (cough/hemoptysis/anemia)	Treatment not reported
27	Masui et al., 2012 (61)	3 mo/F	Right chest wall and eighth rib	Radiograph/CT/MRI showed a multicystic, osteolytic chest-wall mass; mesenchymal hamartoma was favored, with Ewing sarcoma, neuroblastoma, fibrosarcoma, rhabdomyosarcoma, and chondroblastoma also considered	CD68+, CD163+, factor XIIIa focal+; S100−, CD1a−	NR	Mesenchymal hamartoma	Incisional biopsy followed by surgical excision
28	Cevit et al., 2007 (62)	8 y/M	Upper trachea	High-resolution CT and bronchoscopy showed a large polypoid upper-tracheal mass causing marked airway obstruction after treatment as asthma	CD68+ (KP1/PGM1), vimentin+; S100−, CD1a−	NR	Asthma (initially treated as asthma)	Rigid bronchoscopy with endoscopic removal
29	Prakash et al., 2012 (63)	Not reported	Pelvic and retrovesical deep soft tissue	Not reported	Biopsy confirmed JXG	NR	NR	Treatment not reported
30	Heintz et al., 2015 (64)	Not reported	Pancreatic head	Not reported	Biopsy confirmed JXG	NR	NR (pancreatic head mass)	Treatment not reported
31	Ruiz et al., 2008 (65)	6 mo/M	Paratesticular mass	Paratesticular rhabdomyosarcoma considered	Intraoperative frozen section and final pathology confirmed JXG	NR	Paratesticular rhabdomyosarcoma	Intraoperative frozen section and testis-sparing tumorectomy
32	Mitra et al., 2016 (66)	Not reported	Kidney	Not reported	Nephrectomy specimen confirmed JXG	NR	NR (renal mass)	Treatment not reported
33	Dincaslan et al., 2008 (67)	Not reported	Deep axillary soft tissue	Not reported	Resection specimen confirmed giant JXG	NR	NR (vascular mass suggested)	Treatment not reported
34	Seng et al., 2016 (68)	Infant/NR	Cardiac mass with pleural nodules	Not reported	Biopsy confirmed JXG	NR	NR	LCH-based chemotherapy
35	Hara et al., 2006 (69)	2 mo at onset/M	Bone marrow, multisystem	Fever, anaemia, and hepatosplenomegaly; HLH or juvenile myelomonocytic leukaemia considered	Bone marrow and tissue pathology confirmed JXG	NR	HLH or JMML	Etoposide; vinblastine plus prednisolone
36	Jain et al., 2013 (70)	Not reported	Solitary CNS lesion	Diagnostic impression not reported	Histopathology confirmed JXG	NR	Choroid plexus tumor	Treatment not reported
37	Verma et al., 2022 (71)	Not reported	Subglottis	Not reported	Biopsy confirmed JXG (case letter)	NR	NR	Treatment not reported
38	Matcham et al., 2007 (72)	Child/NR	Abdominal wall muscle and lung, multisystem	CT suggested a sarcoma with pulmonary metastases	Biopsy confirmed JXG (abstract)	NR	Sarcoma with pulmonary metastases	Treatment not reported
39	Takeuchi et al., 2009 (73)	Newborn/NR	Congenital multisystem JXG with placental lesion	Not reported	Skin biopsy confirmed systemic JXG; placental infiltrates CD68+, factor XIIIa+	NR	NR	Treatment not reported
40	Lu et al., 2020 (74) Database: CNKI	1 mo 17 d/M	Trunk muscle: right iliolumbar abdominal wall and left lower-rib soft tissue	Intramuscular trunk mass; fibrosarcoma considered	Biopsy-confirmed JXG	NR	Fibrosarcoma	Local excision
41	Dai et al., 2019 (75) Database: CNKI	4 y/F	Palatal submucosa	Palatal submucosal mass; mucoepidermoid carcinoma considered	Biopsy-confirmed JXG	NR	Mucoepidermoid carcinoma	Local excision
42	Ishiyama et al., 2012 (76) Database: Embase	Not reported	Peritoneum and omentum, multisystem	Ultrasound/CT showed heterogeneous peritoneal-based masses involving the omentum with liver scalloping; MRI showed mildly T2-hyperintense masses	Biopsy confirmed JXG	NR	Peritoneal metastasis/RMS/DSRCT	Treatment not reported
43	Dawoud et al.,2019 (77)Database: Embase	18 mo/M	Liver, multisystem	Ultrasound/CT showed hepatomegaly with numerous solid, hypoechoic/hypodense liver lesions; infection, neoplasm, or metastatic disease considered	Percutaneous liver biopsy confirmed JXG	NR	Metastatic disease (imaging)	LCH-type chemotherapy after JXG diagnosis

This table presents 43 representative literature cases and is not the full patient-level evidence set. “Treatment not reported” indicates that treatment details were not available in the accessible primary report; it does not indicate watch-and-wait management.

The two reports retained from CNKI described malignancy-mimicking presentations: an intramuscular trunk lesion in a 1-month-old boy initially suspected to be fibrosarcoma, and a palatal submucosal lesion in a 4-year-old girl initially suspected to be mucoepidermoid carcinoma. Clinical severity ranged from localized, surgically manageable masses to vision-threatening ocular disease, CNS lesions, and life-threatening multisystem disease. The principal diagnostic challenges were unusual anatomical sites, tumor-like imaging findings, variable histological features, and overlap with other histiocytic disorders. Follow-up duration was explicitly reported for 4 of the 15 patients with a malignancy-mimicking presentation (median, 3.5 months; range, 2–48). Study quality was appraised with the JBI critical appraisal checklists for all included reports; per-study ratings are provided in **Supplementary Table S5**.

## 4 Discussion

This study reports five institutional cases of pediatric extracutaneous JXG and integrates them with a PRISMA 2020-guided systematic review of PubMed/MEDLINE, Embase, and CNKI (2006–2026), comprising 147 unique patients. The institutional cases arose in uncommon anatomical sites: the chest wall and pleural cavity, spinal epidural space, Meckel’s cave, kidney, and pineal region. The reviewed literature extends this anatomical range to include the CNS, orbit and ocular adnexa, lung, liver, kidney, bone, pancreas, airway, salivary gland, and deep soft tissue (12–14, 16, 17). Previous reviews have summarized the clinical spectrum and treatment of extracutaneous JXG (12, 13). The present review contributes a patient-level analysis of pre-pathology malignancy-mimicking presentations (15/147, 10.2%) and their consequences (11/15, 73.3%). Across these sites, extracutaneous JXG manifests as a clinicopathological spectrum — varying in cellularity, immunophenotype, and molecular profile — rather than a single stereotyped lesion. CNS lesions may mimic glioma, meningioma, lymphoma, germ cell tumor, metastasis, granulomatous inflammation, or chronic subdural hematoma. Renal lesions may mimic Wilms tumor or renal cell carcinoma. Pancreatic, pulmonary, airway, and chest wall lesions may suggest epithelial, mesenchymal, or small round cell malignancy (18–21). Ocular lesions may mimic melanoma, uveitis, or vascular lesions (22–27). When imaging raises concern for malignancy, rarer diagnoses such as JXG should be included in the clinical and histological differential diagnosis. Biopsy or resection remains essential to establish the diagnosis, and it also serves to spare the child unnecessary oncological and surgical treatment and to optimize the initial therapeutic approach: watch-and-wait can be an option in extracutaneous JXG when the case is eligible.

### 4.1 Diagnostic evidence

Histopathology remains the diagnostic anchor. Typical JXG contains mononuclear histiocytes, foamy histiocytes, variable eosinophils, and Touton-type giant cells. Deep or early lesions, however, may be highly cellular, Touton-poor, or cytologically atypical, explaining why some cases are initially suspected to be sarcoma, myeloid neoplasm, glioma, meningioma, melanoma, Wilms tumor, or another malignancy. These cases illustrate the core principle that histopathological diagnosis requires integration of architecture with immunophenotype; reliance on any single feature invites misclassification. Immunohistochemistry should be interpreted as a coordinated panel, not as individual markers read in isolation. The most reproducible profile is CD68/CD163 positivity with absence of CD1a and langerin, which helps separate JXG from Langerhans cell histiocytosis. Focal S100, CD4, or variable factor XIIIa staining may occur and should not exclude JXG when morphology and the broader immunophenotype are supportive. Difficult cases should be distinguished from Langerhans cell histiocytosis, Rosai–Dorfman–Destombes disease, ALK-positive histiocytosis, pediatric Erdheim–Chester disease, histiocytic sarcoma, inflammatory myofibroblastic tumor, myeloid sarcoma, and site-specific pediatric malignancies; case-by-case exclusion in our cases is detailed in **Supplementary Table S3**.

Molecular findings should be interpreted cautiously. No molecular alteration identified to date is specific to or diagnostic of JXG; diagnosis therefore remains primarily morphologic and immunophenotypic (28). Reported events include BRAF V600E, MAP2K1 and other RAS-MAPK pathway alterations, ALK rearrangements, NTRK fusions or expression, MYH9::FLT3 fusion, CLTC::SYK fusions, CSF1R mutations, and other kinase pathway abnormalities (6–11, 23, 29–36). These findings are biologically relevant, but none is pathognomonic for conventional JXG, and none—including PIK3CA p.H1047R—predicts therapeutic response; treatment decisions based on molecular findings require prospective validation. They instead support a clonal subset of JXG-family lesions driven by MAPK/ERK or related kinase pathways. The PIK3CA p.H1047R mutation in Case 1 should be viewed in this context. This activating hotspot promotes PI3K-AKT-mTOR signaling, and mutant PIK3CA can act as a driver in histiocytic neoplasms (37); genomic alterations involving PIK3CD have been reported in JXG (38). The low variant allele frequency (1.96%) should not be overinterpreted; it may reflect low lesional cell content, inflammatory admixture, subclonality, or formalin-fixed tissue effects. The finding is compatible with PI3K-AKT-mTOR activation, but given the low variant allele frequency and absence of orthogonal validation, it does not by itself establish clonality, confirm JXG, or demonstrate therapeutic actionability.

Molecular testing is most useful when it answers a clinical question. It may be considered for CNS disease, multifocal or systemic disease, recurrence, unresectable tumors, atypical histology, or cases in which targeted therapy is being considered. BRAF V600E immunohistochemistry may serve as a screening test, but sequencing-based confirmation is preferable before targeted treatment is considered. ALK, pan-TRK, and broader DNA/RNA profiling may also be valuable in systemic, infantile, soft-tissue, CNS, or refractory disease. Based on small, heterogeneous case-level literature and institutional experience, we outline practical diagnostic considerations rather than an evidence-based standard: consider JXG in a child with a deep, visceral, ocular, or CNS mass when histology does not fit a malignant radiological impression; review morphology for histiocytic proliferation, xanthomatous change, Touton-type giant cells, eosinophils, and absence of convincing high-grade malignant architecture; use immunohistochemistry to confirm histiocytic differentiation and exclude Langerhans cell, dendritic cell, myeloid, melanocytic, and sarcomatous mimics; and use molecular testing selectively to support clonality, refine classification, or identify potentially targetable alterations. This approach is summarized in **Figure 7**.

**Figure 7:**
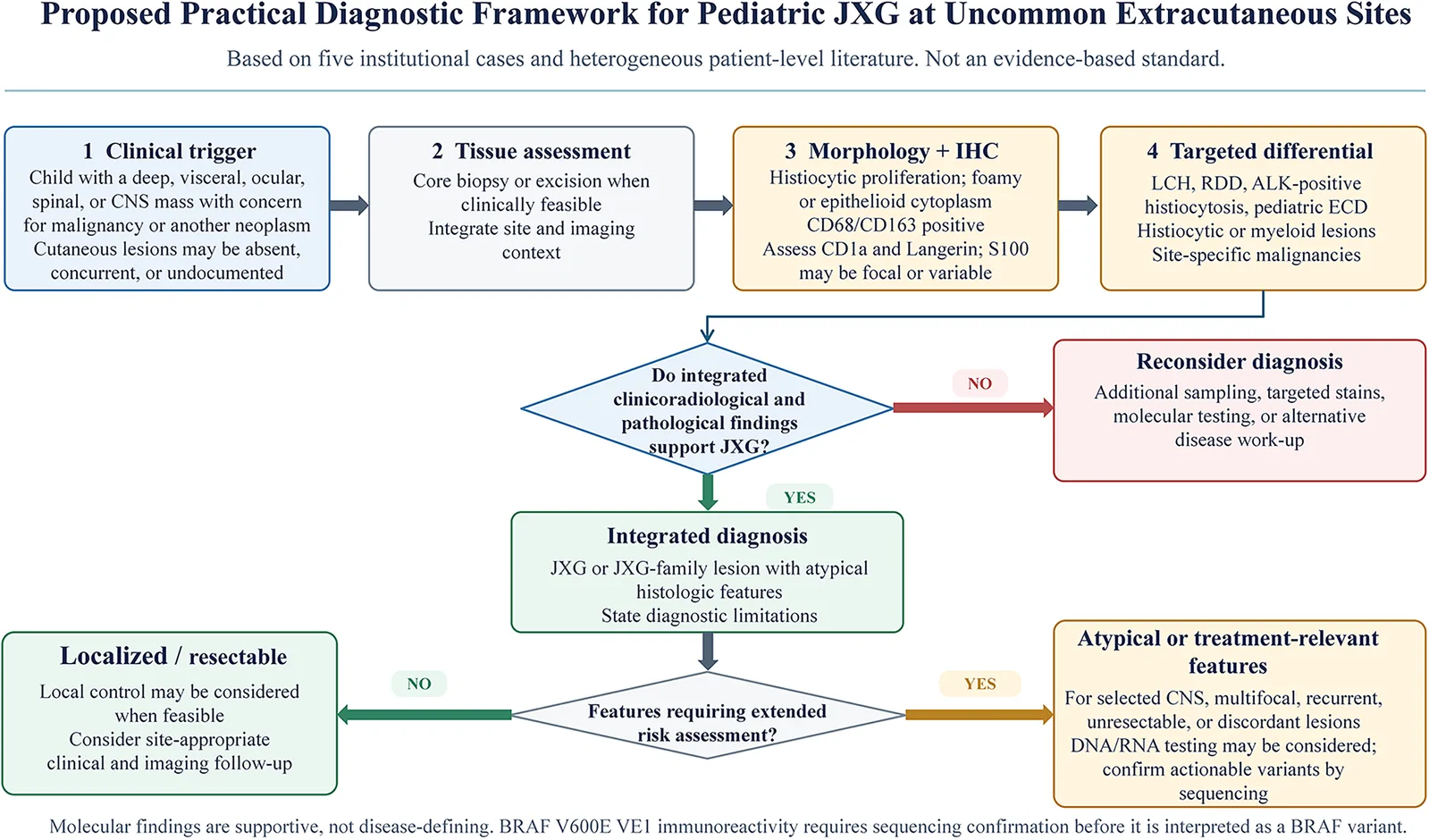
Proposed practical diagnostic framework for pediatric extracutaneous juvenile xanthogranuloma. The framework is based on small, heterogeneous case-level literature and institutional experience and is not intended as an evidence-based diagnostic or management standard. The approach begins with clinical and radiological concern in a child with a deep, visceral, ocular, spinal, or CNS mass, with or without typical cutaneous findings. Tissue diagnosis integrates lesion morphology, immunophenotype, and the site-specific differential diagnosis. Foamy histiocytes and Touton-type giant cells are supportive when present; CD68 and/or CD163 expression supports histiocytic differentiation, whereas CD1a and langerin negativity, when assessed, supports exclusion of Langerhans cell histiocytosis. Additional stains may be selected to address major histiocytic disorders and site-specific malignant mimics. For localized, resectable lesions, surgical management may be considered when clinically feasible. In selected patients with atypical histology, CNS involvement, multifocal, recurrent, unresectable, refractory, or potentially treatment-relevant disease, molecular testing and multidisciplinary evaluation may be considered. Molecular findings may support classification or treatment selection but are not diagnostic of JXG.

### 4.2 Treatment experiences

In our series, complete resection alone was curative in Cases 2–4; Case 1 received low-dose prednisone for locally invasive disease, and Case 5 received Group 2 multiagent chemotherapy for multifocal CNS and calvarial involvement; all five patients remained alive without recurrence during 19–50 months of follow-up. Treatments reported in the literature ranged from observation and surgical excision to corticosteroids, chemotherapy, targeted therapy, and hematopoietic stem cell transplantation. These case-level observations describe current practice patterns and do not constitute treatment recommendations.

This study is limited by its retrospective design, the small institutional cohort, selection of unusually challenging cases referred for expert review, incomplete molecular testing, variable follow-up duration, and heterogeneous case-level reporting. Adjudication of malignancy-mimicking presentation was retrospective. Accordingly, the diagnostic, prognostic, and treatment considerations discussed here derive from case-level observations and should be regarded as descriptive rather than evidence-based recommendations. These observations may inform, but do not define, a practical diagnostic approach integrating site, age, morphology, a core immunohistochemical panel, exclusion of major mimics, and selective molecular testing. Follow-up varied with disease extent, and no standardized surveillance schedule can be derived from the available evidence.

## 5 Conclusion

Pediatric extracutaneous JXG can involve deep soft tissue, viscera, ocular structures, and the CNS, and may mimic malignancy or other tumor-like growth patterns on imaging. Diagnosis requires integration of clinical context, imaging, morphology, immunophenotype, and selective molecular testing. No molecular alteration is specific to or diagnostic of JXG. However, MAPK/ERK or PI3K-AKT-mTOR pathway alterations can identify candidate therapeutic targets in selected cases, provided the finding is orthogonally validated. Recognizing JXG as a clinicopathological spectrum avoids overtreatment of localized disease while identifying patients who require intensified surveillance or systemic therapy. Multicenter studies with standardized molecular profiling and longitudinal follow-up are needed to define genotype-phenotype correlations and establish evidence-based, risk-adapted management.

## Data Availability

The raw data supporting the conclusions of this article will be made available by the authors, without undue reservation.
