# Supplementary material for: Juvenile xanthogranuloma mimicking malignancy at uncommon sites in children: five cases and a systematic review
Source: Front Oncol. 2026 Oct 23;16:1976218. doi: 10.3389/fonc.2026.1976218 (PMC13739496; doi:10.3389/fonc.2026.1976218)
Supplement: Supplementary file 1 [file Table1.docx]

Juvenile Xanthogranuloma Mimicking Malignancy at Uncommon Sites in Children: Five Cases and A Systematic Review

Shan Zhang¹, Weijian Chen¹,Haomiao Zhou¹, Qiuhong Ma², Rong Wen¹, Li Du^3^*

¹ Department of Pathology, Affiliated Children's Hospital, Xiangya School of Medicine, Central South University (Hunan Children's Hospital), Changsha, Hunan 410007, China

² Department of Radiology, Affiliated Children's Hospital, Xiangya School of Medicine, Central South University (Hunan Children's Hospital), Changsha, Hunan 410007, China

^3^ Department of Critical Care Medicine, Hunan Aerospace Hospital, Hunan Normal University, Changsha, Hunan 410205, China

*Correspondence:

Li Du, E-mail: duli@hunnu.edu.cn

Department of Critical Care Medicine, Hunan Aerospace Hospital, Hunan Normal University, Changsha, Hunan 410205, China

Running Title:Pediatric JXG at Uncommon Sites

**Supplementary Table S1. Complete search strategies and database-specific retrieval results.**

| **Database** | **Search strategy** | **Date searched** | **Records retrieved** |
| --- | --- | --- | --- |
| PubMed/MEDLINE | ("Xanthogranuloma, Juvenile"[MeSH Terms] OR "juvenile xanthogranuloma"[Title/Abstract] OR "juvenile xanthogranulomatosis"[Title/Abstract]) AND ("2006/01/01"[Date - Publication] : "2026/09/12"[Date - Publication]) | 12 Sep 2026 | 794 |
| Embase | ('juvenile xanthogranuloma'/exp OR 'juvenile xanthogranuloma':ti,ab,kw OR 'juvenile xanthogranulomatosis':ti,ab,kw) AND [1-1-2006]/sd NOT [13-9-2026]/sd | 12 Sep 2026 | 1,402 |
| CNKI | Topic = (("juvenile xanthogranuloma" — all three Chinese synonyms); Date: 2006-01-01 to 2026-09-12 | 12 Sep 2026 | 107 |
| Sensitivity verification (all three databases) | Disease strategy AND (imaging OR MRI OR CT OR ultrasound OR radiolog*) | 12 Sep 2026 | verification only |

The primary strategy (disease terms only) was used for screening. The combined disease-plus-imaging strategy was used solely to verify search sensitivity and did not contribute records to screening. All searches were executed without language restriction on 12 September 2026 (2006-01-01 to 2026-09-12).

**Supplementary Table S2. Primary antibodies used for immunohistochemistry.**

| **Antibody** | **Clone** | **Host species** | **Supplier** | **Catalogue no.** |
| --- | --- | --- | --- | --- |
| ALK | OTI1H7 | Mouse monoclonal | Beijing Zhongshan Golden Bridge Biotechnology Co., Ltd. (ZSGB-BIO), Beijing, China | ZM-0248 |
| CD163 | 10D6 | Mouse monoclonal | Beijing Zhongshan Golden Bridge Biotechnology Co., Ltd. (ZSGB-BIO), Beijing, China | ZM-0428 |
| CD68 | KP1 | Mouse monoclonal | Beijing Zhongshan Golden Bridge Biotechnology Co., Ltd. (ZSGB-BIO), Beijing, China | ZM-0060 |
| S100 | 15E2E2+4C4.9 | Mouse monoclonal | Beijing Zhongshan Golden Bridge Biotechnology Co., Ltd. (ZSGB-BIO), Beijing, China | ZM-0224 |
| Ki-67 | MIB1 | Mouse monoclonal | Beijing Zhongshan Golden Bridge Biotechnology Co., Ltd. (ZSGB-BIO), Beijing, China | ZM-0167 |
| BRAF V600E | RM8 | Rabbit monoclonal | Beijing Zhongshan Golden Bridge Biotechnology Co., Ltd. (ZSGB-BIO), Beijing, China | ZA-0668 |
| BRG1 (SMARCA4) | E8V5B | Rabbit monoclonal | Beijing Zhongshan Golden Bridge Biotechnology Co., Ltd. (ZSGB-BIO), Beijing, China | ZA-0673 |
| CD1a | EP80 | Rabbit monoclonal | Beijing Zhongshan Golden Bridge Biotechnology Co., Ltd. (ZSGB-BIO), Beijing, China | ZA-0544 |
| CD30 | UMAB256 | Mouse monoclonal | Beijing Zhongshan Golden Bridge Biotechnology Co., Ltd. (ZSGB-BIO), Beijing, China | ZM-0043 |
| CD31 | NR | Mouse monoclonal | Beijing Zhongshan Golden Bridge Biotechnology Co., Ltd. (ZSGB-BIO), Beijing, China | ZM-0044 |
| CD4 | UMAB64 | Mouse monoclonal | Beijing Zhongshan Golden Bridge Biotechnology Co., Ltd. (ZSGB-BIO), Beijing, China | ZM-0418 |
| CD117 | EP10 | Rabbit monoclonal | Beijing Zhongshan Golden Bridge Biotechnology Co., Ltd. (ZSGB-BIO), Beijing, China | ZA-0523 |
| Fascin | IM20 | Mouse monoclonal | Beijing Zhongshan Golden Bridge Biotechnology Co., Ltd. (ZSGB-BIO), Beijing, China | ZM-0238 |
| CD10 | UMAB235 | Mouse monoclonal | Beijing Zhongshan Golden Bridge Biotechnology Co., Ltd. (ZSGB-BIO), Beijing, China | ZM-0283 |
| EMA | GP1.4 | Mouse monoclonal | Beijing Zhongshan Golden Bridge Biotechnology Co., Ltd. (ZSGB-BIO), Beijing, China | ZM-0095 |
| INI-1/SMARCB1 | OTIR4G9 | Rabbit monoclonal | Beijing Zhongshan Golden Bridge Biotechnology Co., Ltd. (ZSGB-BIO), Beijing, China | ZA-0696 |
| TFE3 | EP285 | Rabbit monoclonal | Beijing Zhongshan Golden Bridge Biotechnology Co., Ltd. (ZSGB-BIO), Beijing, China | ZA-0657 |
| H3K27Me3 | RM175 | Rabbit monoclonal | Beijing Zhongshan Golden Bridge Biotechnology Co., Ltd. (ZSGB-BIO), Beijing, China | ZA-0327 |
| Rb | 13A10 | Mouse monoclonal | Beijing Zhongshan Golden Bridge Biotechnology Co., Ltd. (ZSGB-BIO), Beijing, China | ZM-0223 |
| Vimentin | V9 | Mouse monoclonal | Beijing Zhongshan Golden Bridge Biotechnology Co., Ltd. (ZSGB-BIO), Beijing, China | ZM-0260 |
| WT1 | 6F-H2 | Mouse monoclonal | Beijing Zhongshan Golden Bridge Biotechnology Co., Ltd. (ZSGB-BIO), Beijing, China | ZM-0269 |
| CD56 | UMAB83 | Mouse monoclonal | Beijing Zhongshan Golden Bridge Biotechnology Co., Ltd. (ZSGB-BIO), Beijing, China | ZM-0057 |
| PAX8 | OTI6H8 | Mouse monoclonal | Beijing Zhongshan Golden Bridge Biotechnology Co., Ltd. (ZSGB-BIO), Beijing, China | ZM-0468 |
| PGP9.5 | UMAB244 | Mouse monoclonal | Beijing Zhongshan Golden Bridge Biotechnology Co., Ltd. (ZSGB-BIO), Beijing, China | ZA-0263 |
| Pan-cytokeratin | AE1/AE3 | Mouse monoclonal | Beijing Zhongshan Golden Bridge Biotechnology Co., Ltd. (ZSGB-BIO), Beijing, China | ZM-0069 |
| Cytokeratin 8/18 | B22.1+B23.1 | Mouse monoclonal | Beijing Zhongshan Golden Bridge Biotechnology Co., Ltd. (ZSGB-BIO), Beijing, China | ZM-0315 |
| Cyclin D1 | SA38-08 | Rabbit monoclonal | Beijing Zhongshan Golden Bridge Biotechnology Co., Ltd. (ZSGB-BIO), Beijing, China | ZA-0101 |
| Desmin | OTIR4A8 | Rabbit monoclonal | Beijing Zhongshan Golden Bridge Biotechnology Co., Ltd. (ZSGB-BIO), Beijing, China | ZA-0610 |
| CD34 | 10C9 | Mouse monoclonal | Beijing Zhongshan Golden Bridge Biotechnology Co., Ltd. (ZSGB-BIO), Beijing, China | ZM-0046 |
| Myogenin | EP162 | Rabbit monoclonal | Beijing Zhongshan Golden Bridge Biotechnology Co., Ltd. (ZSGB-BIO), Beijing, China | ZA-0592 |
| HMB45 | HMB45 | Mouse monoclonal | Beijing Zhongshan Golden Bridge Biotechnology Co., Ltd. (ZSGB-BIO), Beijing, China | ZM-0187 |
| p53 | DO-7 | Mouse monoclonal | Beijing Zhongshan Golden Bridge Biotechnology Co., Ltd. (ZSGB-BIO), Beijing, China | ZM-0408 |
| MyoD1 | EP212 | Rabbit monoclonal | Beijing Zhongshan Golden Bridge Biotechnology Co., Ltd. (ZSGB-BIO), Beijing, China | ZA-0585 |
| Langerin/CD207 | 12D6 | Mouse monoclonal | Beijing Zhongshan Golden Bridge Biotechnology Co., Ltd. (ZSGB-BIO), Beijing, China | ZM-0385 |
| CD21 | 2G9 | Mouse monoclonal | Beijing Zhongshan Golden Bridge Biotechnology Co., Ltd. (ZSGB-BIO), Beijing, China | ZM-0040 |
| CD35 | EP197 | Rabbit monoclonal | Beijing Zhongshan Golden Bridge Biotechnology Co., Ltd. (ZSGB-BIO), Beijing, China | ZA-0638 |
| CD123 | BR4MS | Mouse monoclonal | Beijing Zhongshan Golden Bridge Biotechnology Co., Ltd. (ZSGB-BIO), Beijing, China | ZM-0423 |
| AAT (α1-antitrypsin) | NR | NR | Beijing Children’s Hospital (external laboratory), Beijing, China | NR |
| pan-TRK (TRK A/B/C) | NR | Rabbit monoclonal | Beijing Zhongshan Golden Bridge Biotechnology Co., Ltd. (ZSGB-BIO), Beijing, China | ZA-0687 |
| SMA | 1A4 | Mouse monoclonal | Beijing Zhongshan Golden Bridge Biotechnology Co., Ltd. (ZSGB-BIO), Beijing, China | ZM-0003 |

**Supplementary Table S3. Case-specific differential diagnostic assessment of the five institutional pediatric JXG cases.**

Note. "Not assessed" indicates that no direct case-specific evidence was available in the source record. "Not favored" indicates diagnostic de-prioritization based on the available morphology, immunophenotype, imaging, expert consultation, or clinical course, and should not be interpreted as complete exclusion.

| **Case** | **Langerhans cell histiocytosis (LCH)** | **ALK-positive histiocytosis** | **Pediatric Erdheim–Chester disease (ECD)** | **Histiocytic sarcoma** | **Myeloid neoplasm / myeloid sarcoma** | **Site-specific malignancy** |
| --- | --- | --- | --- | --- | --- | --- |
| Case 1 Chest wall / pleural cavity | Foamy and mononuclear histiocytes with multinucleated giant cells; CD68/CD163 positive; langerin and CD1a negative; CD123 and CD30 negative; targeted NGS identified no BRAF alteration. LCH was not favored. | ALK IHC negative; targeted NGS identified no ALK alteration. Not favored. A DNA-based panel does not formally exclude ALK rearrangements (RNA-based fusion testing was not performed). | No characteristic long-bone imaging pattern or disease-defining molecular result documented. Not assessed. | Mass-forming lesion with focal necrosis and calcification; Ki-67 approximately 20%; histiocytic markers positive. Not assessed; no dedicated high-grade morphology assessment was documented. | Bone marrow examination: hypercellular marrow with active granulopoiesis, decreased erythropoiesis, and no morphologic abnormality; MPO negative; CD34 highlighted vessels only. Not favored by the available marrow morphology. Flow cytometry was not documented. | Imaging initially favored Askin tumor, pleuropulmonary blastoma, or PNET. SATB2, myogenin, SALL4, and calretinin were negative; LCA negative; CK and desmin focally positive; MyoD1 rarely positive; INI1 retained. Focal weak CD99 and NKX2.2 positivity was noted but was not considered sufficient for a diagnosis of Ewing-family tumor (no EWSR1 rearrangement testing documented); TFE3 was positive (no rearrangement testing). Targeted NGS identified no BRAF, NTRK1, NTRK2, NTRK3, or RET variant. Radiologic malignancy was not favored after pathologic review. |
| Case 2 Spinal epidural space | Histiocytic proliferation with inflammatory cells; CD68/CD163/CD4 positive, scattered S100 positive; CD1a and langerin negative. Not favored. | ALK IHC negative. Not favored. No ALK rearrangement test was documented; a negative stain alone is not molecular exclusion. | No characteristic long-bone imaging pattern or defining molecular result. Not assessed. | Ki-67 approximately 20%; no documented high-grade architecture or atypical mitotic activity. Not assessed; no dedicated histiocytic sarcoma panel was available. | No MPO, CD34, or flow cytometry; bone marrow examination was not performed. Not assessed. | Initially interpreted radiologically as a nerve sheath tumor (spinal cord compression); histiocytic immunophenotype documented. Nerve sheath tumor was considered less likely after histopathologic review. No tumor-specific neural or schwannian marker panel was available. |
| Case 3 Meckel's cave | Mononuclear and foamy histiocytes with inflammatory cells; CD68/CD163/CD4 positive; CD1a and langerin negative. Not favored (strongest LCH exclusion evidence among the five cases). | No ALK IHC or rearrangement result available. Not assessed. | No characteristic long-bone imaging pattern or defining molecular result. Not assessed. | Ki-67 approximately 5%; no high-grade malignant architecture documented. Not favored by the recorded morphology and low proliferative index, but not formally excluded; no dedicated panel was available. | No MPO, CD34, or flow cytometry; bone marrow examination was not performed. Not assessed. | Radiologically considered a trigeminal neuroma or meningioma; histiocytic morphology and immunophenotype supported JXG. The radiologic alternatives were not favored after pathology review. No meningioma or schwannian marker panel was available. |
| Case 4 Kidney | Markedly pleomorphic histiocytic lesion with bizarre nuclei and multifocal necrosis; CD163/CD4/fascin positive; CD1a and langerin negative. Not favored. | ALK IHC negative; targeted sequencing identified no pathogenic alteration. Not favored. No ALK rearrangement test was documented; a negative stain alone is not molecular exclusion. | No symmetric long-bone osteosclerosis, characteristic systemic imaging pattern, or defining molecular result documented. Not assessed. | Marked pleomorphism, bizarre nuclei, and multifocal necrosis prompted serious consideration; expert consultation at Beijing Children's Hospital favored a JXG-family lesion; disease-free approximately 34 months after surgery alone. Not favored after integrated immunophenotypic review, expert consultation, and clinical course; the wording should remain "not favored," not "ruled out." | CD34 stained vessels only; MPO and flow cytometry were not performed. Could not be fully excluded by direct myeloid lineage testing. | Imaging initially favored Wilms tumor. WT1, PAX8, CK, CK8/18, CD10, CD56, desmin, myogenin, MyoD1, HMB45, cyclin D1, p53, and TRK were negative; EMA and PGP9.5 focally positive; INI1, BRG1, H3K27me3, and Rb retained; TFE3 weakly positive. Wilms tumor, renal epithelial tumors, rhabdomyogenic neoplasms, and PEComa were not favored on the recorded panel. Weak TFE3 positivity warrants caution regarding a MiT-family translocation renal cell carcinoma; rearrangement testing was not documented. |
| Case 5 Pineal region | Foamy histiocytes in a fibroinflammatory background; CD68/CD163 positive, S100 diffusely positive; CD1a negative. Not favored. Langerin was not tested; its absence should not be inferred. | ALK IHC negative. Not favored. No ALK rearrangement test was documented. | Multifocal CNS and calvarial lesions recorded; no characteristic long-bone pattern or defining molecular result. Not assessed. | Ki-67 approximately 1%; foamy histiocytes in a fibroinflammatory background. Not favored by the recorded morphology and low proliferative index, but not formally excluded. | Bone marrow examination: active marrow with decreased granulopoiesis, prominent erythropoiesis, and no morphologic abnormality. Not favored by the available marrow morphology. MPO and flow cytometry were not documented. | Pineoblastoma and germinoma were considered radiologically; lin28 and c-KIT negative; histiocytic morphology with CD68/CD163 positivity supported JXG. Germinoma was considered less likely; pineoblastoma was not formally excluded by a documented tumor-specific panel. |

Abbreviations: ECD, Erdheim–Chester disease; IHC, immunohistochemistry; JXG, juvenile xanthogranuloma; LCH, Langerhans cell histiocytosis; MiT, microphthalmia-associated transcription factor; NGS, next-generation sequencing; PEComa, perivascular epithelioid cell tumor

**Supplementary Table S4. Patient-level overlap resolution.**

Overlapping patients reported in multiple publications were identified using a predefined patient fingerprint comprising first and corresponding author, institution, publication year, age, sex, exact anatomical site, lesion multiplicity, clinical presentation, imaging features, treatment, follow-up, and molecular findings. Record-level deduplication before screening (674 duplicates: 662 between PubMed/MEDLINE and Embase and 12 within Embase) is a separate step and is not listed here. Each overlap pair was classified and resolved as shown; all listed pairs had zero net effect on patient-unit counting.

| **No.** | **Record A** | **Record B** | **Overlap evidence (fingerprint)** | **Decision** | **Retained record** | **Patient-unit impact** |
| --- | --- | --- | --- | --- | --- | --- |
| 1 | E0154 + E0156 (cardiac JXG, conference abstracts) | P40250921 (full report; JACC Case Rep 2025) | Same patient (cardiac JXG) | Merged; abstracts not counted independently | P40250921 | 0 |
| 2 | E1017 (Embase record) | E1092 — Wille et al., Neuropediatrics 2013 (PMID 23254572) | Same primary report (PubMed/Embase records of one publication) | Single report-level unit retained | Wille et al., 2013 (PMID 23254572) | 0 |
| 3 | E0233 (head-and-neck series, 11 cases) | P38832444 (full report) | Series–full report overlap; series cases assessed per patient | Linked at patient level | P38832444 | 0 |
| 4 | CN26 (CNKI) | P34178890 (English-language report) | Same patient | Patient-level overlap; CNKI record not counted as an independent record | P34178890 | 0 |
| 5 | CN39 (CNKI) | P32981400 (English-language report) | Same patient | Patient-level overlap; CNKI record not counted as an independent record | P32981400 | 0 |

† Neither record contributes a patient unit in the main-pool roster; the pair was resolved at the record level and does not change the patient count.

Reconciliation of report and patient counts. The 150 included reports comprised 134 main-pool reports and 16 supplementary reports. Patient-level linkage yielded 137 unique patients from the main pool, with per-patient expansion applied to multi-patient series reports, and 10 unique patients from the supplementary pool (six independent candidates, one candidate re-adjudicated as independent after fingerprint review, two patients from supplementary record #86, and one patient from supplementary record #130). The difference between the 150 included reports and the 147 unique patients therefore reflects the combined effect of per-patient expansion of multi-patient series within the main pool and patient-level deduplication within the supplementary pool, rather than a single set of duplicate-report pairs; the five overlap pairs listed above had zero net effect on patient-unit counting.

**Supplementary Table S5. JBI critical appraisal of included case reports and case series (138 primary-report units).**

Assessment unit: primary report (not individual patient); multiple patients within one report share a single report-level appraisal. Critical appraisal was based on the fullest accessible source for each report; domains that could not be verified from the available source were rated as unclear. Case reports and letters with case-level data were assessed with the eight-item JBI Critical Appraisal Checklist for Case Reports (Q1–Q8; Q9–Q10 left blank); case series with the ten-item JBI Critical Appraisal Checklist for Case Series (Q1–Q10). Q7 (adverse or unanticipated events) was rated Yes when such events were explicitly reported or explicitly stated to be absent, and Unclear when not addressed. Conference abstracts and posters were appraised only on the domains verifiable from that source. Responses: Yes / No / Unclear / Not applicable; Unclear indicates insufficient reporting or source access, not a negative finding. No numerical quality score was calculated; no missing information was imputed as Yes.

| **No.** | **Study** | **Year** | **PMID** | **Study design** | **Q1** | **Q2** | **Q3** | **Q4** | **Q5** | **Q6** | **Q7** | **Q8** | **Q9** | **Q10** | **Source** | | **Overall appraisal** |
| --- | --- | --- | --- | --- | --- | --- | --- | --- | --- | --- | --- | --- | --- | --- | --- | --- | --- |
| 1 | Dawoud M (2019) | 2019 | — | Conference poster/case report | Y | Y | Y | Y | Y | Y | U | Y |  |  | | ABS | Include with reporting limitations |
| 2 | Hyphaema: A diagnostic dilemma | — | — | Conference abstract | Y | U | Y | Y | Y | Y | U | Y |  |  | | ABS | Include with reporting limitations |
| 3 | Areal-Hidalgo P (2018) | 2018 | — | Case report | Y | Y | Y | Y | Y | Y | Y | Y |  |  | | FT | Include |
| 4 | Myeong HS (2021) | 2021 | — | Conference abstract | U | U | U | U | U | U | U | U |  |  | | ABS | Not appraisable |
| 5 | Shanmugam S (2020) | 2020 | 33342885 | Case report | Y | U | Y | Y | Y | Y | U | Y |  |  | | ABS | Include |
| 6 | Yang J (2021) | 2021 | 33771776 | Case report | Y | Y | Y | Y | Y | Y | U | Y |  |  | | FT | Include |
| 7 | Hara T (2006) | 2006 | 16047346 | Case report | Y | Y | Y | Y | Y | Y | U | Y |  |  | | ABS | Include |
| 8 | Wang SN (2022) | 2022 | 35151291 | Case report | Y | Y | Y | Y | Y | Y | U | Y |  |  | | FT | Include |
| 9 | Maejima A (2024) | 2024 | 39603632 | Case report | Y | Y | Y | Y | Y | Y | U | Y |  |  | | FT | Include |
| 10 | Fontanilla FA (2009) | 2009 | 19840735 | Case report | Y | U | Y | Y | Y | Y | U | Y |  |  | | ABS | Include |
| 11 | Matsunaga D (2020) | 2020 | 32621982 | Case report | Y | U | Y | Y | Y | Y | U | Y |  |  | | ABS | Include |
| 12 | Matcham NJ (2007) | 2007 | 17230073 | Case report | Y | U | Y | Y | Y | Y | U | Y |  |  | | ABS | Include |
| 13 | Ruiz E (2008) | 2008 | 18538690 | Case report | Y | Y | Y | Y | Y | Y | U | Y |  |  | | ABS | Include |
| 14 | Kwok C (2015) | 2015 | 25787311 | Case report | Y | U | Y | Y | Y | U | U | Y |  |  | | ABS | Include |
| 15 | Sagga A (2023) | 2023 | 37285582 | Case report | Y | Y | Y | Y | Y | Y | U | Y |  |  | | FT | Include |
| 16 | Irmola T (2018) | 2018 | 29876657 | Case report | Y | U | Y | Y | Y | Y | U | Y |  |  | | ABS | Include |
| 17 | Yu L (2020) | 2020 | — | Case report | Y | U | Y | Y | U | U | U | Y |  |  | | ABS | Include with reporting limitations |
| 18 | Lu T (2020) | 2020 | — | Case report | Y | U | Y | Y | Y | U | U | Y |  |  | | ABS | Include with reporting limitations |
| 19 | Peng X (2020) | 2020 | — | To verify | U | U | U | U | U | U | U | U |  |  | | — | Seek further information |
| 20 | Dai B (2019) | 2019 | — | Case report | Y | U | Y | Y | Y | Y | U | Y |  |  | | ABS | Include |
| 21 | Liang S (2008) | 2008 | — | To verify | U | U | U | U | U | U | U | U |  |  | | — | Seek further information |
| 22 | Ito J (2019) | 2019 | 31309704 | Case report | Y | U | Y | Y | Y | Y | U | Y |  |  | | ABS | Include |
| 23 | Wen Z (2020) | 2020 | 32049719 | Case report | Y | U | Y | Y | Y | Y | U | Y |  |  | | ABS | Include |
| 24 | Myeong HS (2021) | 2021 | 33660104 | Case report | Y | U | Y | Y | Y | U | U | Y |  |  | | ABS | Include |
| 25 | Leung WY (2026) | 2026 | 41935961 | Case report | Y | Y | Y | Y | Y | U | U | Y |  |  | | ABS | Include |
| 26 | Däniker M (2026) | 2026 | 41170802 | Case series | U | Y | Y | U | U | Y | Y | Y | Y | N | | FT | Include with reporting limitations |
| 27 | Irie R (2022) | 2022 | 34840207 | Case series | U | Y | Y | U | U | Y | Y | Y | Y | N | | FT | Include with reporting limitations |
| 28 | Toker M (2024) | 2024 | 38444069 | Case series | U | Y | Y | U | U | Y | Y | Y | Y | N | | ABS | Include with reporting limitations |
| 29 | Mavi ME (2022) | 2022 | 35085175 | Case report | Y | Y | Y | Y | Y | Y | U | Y |  |  | | ABS | Include |
| 30 | Zhang C (2021) | 2021 | 34288768 | Case report | Y | Y | Y | Y | Y | Y | U | Y |  |  | | REC | Include |
| 31 | Sako M (2020) | 2020 | 32507810 | Case report | Y | Y | Y | Y | Y | Y | Y | Y |  |  | | FT | Include |
| 32 | Mamtani N (2025) | 2025 | 40666774 | Case report | Y | Y | Y | Y | Y | Y | U | Y |  |  | | FT | Include |
| 33 | Lee MH (2024) | 2024 | 38687290 | Case series | Y | Y | Y | U | U | Y | Y | Y | Y | N | | ABS | Include with reporting limitations |
| 34 | Larranaga Cores M (2025) | 2025 | 40780441 | Case report | Y | Y | Y | Y | Y | Y | U | Y |  |  | | ABS | Include |
| 35 | Ataseven E (2021) | 2021 | 33998710 | Case report | Y | U | Y | Y | Y | Y | U | Y |  |  | | ABS | Include |
| 36 | Uehara Y (2021) | 2021 | 33823829 | Case report | Y | Y | Y | Y | Y | Y | U | Y |  |  | | FT | Include |
| 37 | Clark EE (2023) | 2023 | 36816441 | Case report | Y | U | Y | Y | Y | Y | U | Y |  |  | | ABS | Include |
| 38 | Singh K (2024) | 2024 | 39150359 | Case report | Y | U | Y | Y | Y | U | U | Y |  |  | | ABS | Include |
| 39 | He S (2020) | 2020 | 32981400 | Case report | Y | Y | Y | Y | Y | Y | U | Y |  |  | | FT | Include |
| 40 | Bilgin E (2021) | 2021 | 33735896 | Case report | Y | Y | Y | Y | Y | Y | U | Y |  |  | | ABS | Include |
| 41 | Lalitha P (2011) | 2011 | 20671061 | Case report | Y | Y | Y | Y | U | U | U | Y |  |  | | FT | Include |
| 42 | Matsubara K (2016) | 2016 | 27435377 | Case report | Y | U | Y | Y | Y | U | U | Y |  |  | | ABS | Include |
| 43 | Gressot LV (2013) | 2013 | 23790105 | Case report | Y | Y | Y | Y | Y | Y | U | Y |  |  | | ABS | Include |
| 44 | Cornips EM (2009) | 2009 | 19951050 | Case report | Y | U | Y | Y | Y | U | U | Y |  |  | | ABS | Include |
| 45 | Seng MS (2017) | 2017 | — | Case report | Y | Y | Y | Y | Y | Y | U | Y |  |  | | ABS | Include |
| 46 | Ishiyama A (2012) | 2012 | — | Case report | Y | U | Y | Y | U | U | U | Y |  |  | | ABS | Include with reporting limitations |
| 47 | Deisch JK (2013) | 2013 | 22640164 | Case series | Y | Y | Y | U | U | Y | Y | Y | Y | N | | ABS | Include with reporting limitations |
| 48 | Mocan MC (2008) | 2008 | 18580271 | Case series | Y | Y | Y | U | U | Y | Y | U | Y | N | | ABS | Include with reporting limitations |
| 49 | De Keyser C (2011) | 2011 | 21174524 | Case series | Y | Y | Y | U | U | Y | Y | U | Y | N | | ABS | Include with reporting limitations |
| 50 | Nakasu S (2007) | 2007 | 17332947 | Case report | Y | Y | Y | Y | Y | Y | U | Y |  |  | | ABS | Include |
| 51 | Johnson TE (2010) | 2010 | 20305521 | Case report | Y | Y | Y | Y | Y | Y | U | Y |  |  | | REC | Include |
| 52 | De Paula AM (2010) | 2010 | 20582967 | Case report | Y | U | Y | Y | Y | Y | U | Y |  |  | | ABS | Include |
| 53 | Yamada K (2012) | 2012 | 21384540 | Case report | Y | Y | Y | Y | Y | Y | Y | Y |  |  | | ABS | Include |
| 54 | Wille DA (2013) | 2013 | 23254572 | Case report | Y | Y | Y | Y | Y | Y | U | Y |  |  | | ABS | Include |
| 55 | Ferguson SD (2015) | 2015 | 25503249 | Case report | Y | Y | Y | Y | Y | Y | Y | Y |  |  | | ABS | Include |
| 56 | Shenoy A (2018) | 2018 | 29492155 | Case report | Y | Y | Y | Y | Y | Y | U | Y |  |  | | FT | Include |
| 57 | Su J (2019) | 2019 | 30820641 | Case report | Y | U | Y | Y | U | U | U | Y |  |  | | ABS | Include with reporting limitations |
| 58 | Ito H (2020) | 2020 | — | Case report | Y | Y | Y | Y | Y | Y | U | Y |  |  | | FT | Include |
| 59 | Kahn JA (2020) | 2020 | 32679560 | Case report | Y | Y | Y | Y | Y | Y | U | Y |  |  | | ABS | Include |
| 60 | Calderon C (2021) | 2021 | 34388899 | Case report | Y | Y | Y | Y | Y | Y | Y | Y |  |  | | FT | Include |
| 61 | Damodharan S (2022) | 2022 | 35304626 | Case report | Y | Y | Y | Y | Y | Y | Y | Y |  |  | | ABS | Include |
| 62 | Rios IS (2023) | 2023 | 37482184 | Case report | Y | Y | Y | Y | Y | Y | U | Y |  |  | | ABS | Include |
| 63 | De Santiago J (2006) | 2006 | 16918661 | Case report | Y | Y | Y | Y | Y | Y | Y | Y |  |  | | ABS | Include |
| 64 | Kesserwan C (2007) | 2007 | 17378689 | Case report | Y | Y | Y | Y | Y | Y | U | Y |  |  | | ABS | Include |
| 65 | Li HC (2008) | 2008 | 18833826 | Case report | Y | Y | Y | Y | Y | U | U | Y |  |  | | ABS | Include |
| 66 | Papadakis V (2012) | 2012 | 22031119 | Case report | Y | Y | Y | Y | Y | Y | U | Y |  |  | | ABS | Include |
| 67 | Masui D (2012) | 2012 | 22864545 | Case report | Y | Y | Y | Y | Y | Y | U | Y |  |  | | FT | Include |
| 68 | Bhaisora KS (2015) | 2015 | 25767592 | Case report | Y | Y | Y | Y | Y | Y | U | Y |  |  | | FT | Include |
| 69 | Cheon E (2018) | 2018 | 28836893 | Case report | Y | Y | Y | Y | Y | Y | U | Y |  |  | | ABS | Include |
| 70 | Rajasekhar R (2019) | 2019 | 30771048 | Case report | Y | Y | Y | Y | Y | Y | U | Y |  |  | | ABS | Include |
| 71 | Zhu J (2026) | 2026 | 41560073 | Case report | Y | Y | Y | Y | Y | Y | U | Y |  |  | | FT | Include |
| 72 | Vendal Z (2006) | 2006 | 16912017 | Case report | Y | U | Y | Y | Y | U | U | Y |  |  | | ABS | Include |
| 73 | Chami R (2010) | 2010 | 21055524 | Case report | Y | U | Y | Y | Y | U | U | Y |  |  | | ABS | Include |
| 74 | Gohari AR (2010) | 2010 | 25390914 | Case report | Y | Y | Y | Y | Y | Y | U | Y |  |  | | ABS | Include |
| 75 | Tsimpida M (2013) | 2013 | 23598670 | Case report | Y | Y | Y | Y | Y | Y | U | Y |  |  | | FT | Include |
| 76 | Manjandavida FP (2014) | 2014 | 24568997 | Case report | Y | Y | Y | Y | Y | Y | U | Y |  |  | | ABS | Include |
| 77 | Rao A (2014) | 2014 | 24928931 | Case report | Y | Y | Y | Y | Y | Y | U | Y |  |  | | FT | Include |
| 78 | Syed ZA (2016) | 2016 | 27483333 | Case report | Y | Y | Y | Y | U | U | U | Y |  |  | | ABS | Include with reporting limitations |
| 79 | Pantalon A (2017) | 2017 | 29450403 | Case report | Y | Y | Y | Y | Y | Y | Y | Y |  |  | | FT | Include |
| 80 | Ramos Suárez A (2017) | 2017 | 28522113 | Case report | Y | Y | Y | Y | Y | Y | U | Y |  |  | | ABS | Include |
| 81 | Nassereddine H (2020) | 2020 | — | Case report | Y | Y | Y | Y | U | U | U | Y |  |  | | ABS | Include with reporting limitations |
| 82 | Mazhar D (2020) | 2020 | 34756387 | Case report | Y | U | Y | Y | U | U | U | Y |  |  | | ABS | Include with reporting limitations |
| 83 | Anilkumar SE (2023) | 2023 | 37252160 | Case report | Y | Y | Y | Y | Y | Y | U | Y |  |  | | FT | Include |
| 84 | Winter AW (2022) | 2022 | 34077746 | Case report | Y | U | Y | Y | Y | Y | U | Y |  |  | | ABS | Include |
| 85 | Esen Baris M (2022) | 2022 | 34228593 | Case report | Y | Y | Y | Y | Y | Y | U | Y |  |  | | ABS | Include |
| 86 | Dericioglu V (2022) | 2022 | 36128087 | Case report | Y | Y | Y | Y | Y | Y | Y | Y |  |  | | FT | Include |
| 87 | Kong XY (2022) | 2022 | 36220652 | Case report | Y | Y | Y | Y | Y | Y | U | Y |  |  | | REC | Include |
| 88 | Suraneni S (2024) | 2024 | 39822764 | Case report | Y | Y | Y | Y | Y | Y | U | Y |  |  | | FT | Include |
| 89 | Boseley ME (2006) | 2006 | — | Case report | Y | Y | Y | Y | Y | U | U | Y |  |  | | FT | Include with reporting limitations |
| 90 | Snijders D (2007) | 2007 | 17647288 | Case report | Y | Y | Y | Y | Y | U | U | Y |  |  | | ABS | Include |
| 91 | Cevit O (2007) | 2007 | — | Case report | Y | Y | Y | Y | Y | Y | U | Y |  |  | | REC | Include |
| 92 | Somorai M (2007) | 2007 | 17123317 | Case report | Y | Y | Y | Y | Y | Y | U | Y |  |  | | ABS | Include |
| 93 | Kawamoto A (2013) | 2013 | 23801051 | Case report | Y | Y | Y | Y | Y | Y | Y | Y |  |  | | ABS | Include |
| 94 | Sosnowska P (2020) | 2020 | 33235547 | Case report | Y | Y | Y | Y | Y | Y | U | Y |  |  | | FT | Include |
| 95 | Janjic O (2026) | 2026 | 42507494 | Case report | Y | Y | Y | Y | Y | Y | Y | Y |  |  | | FT | Include |
| 96 | Ferraz R (2026) | 2026 | 41842676 | Case report | Y | Y | Y | Y | Y | Y | U | Y |  |  | | ABS | Include |
| 97 | Kobayashi D (2013) | 2013 | 22691125 | Case report | Y | Y | Y | Y | Y | Y | U | Y |  |  | | ABS | Include |
| 98 | Mehdi MQ (2025) | 2025 | 40250921 | Case report | Y | Y | Y | Y | Y | Y | U | Y |  |  | | FT | Include |
| 99 | Weiss VL (2014) | 2014 | 24582118 | Case report | Y | Y | Y | Y | Y | U | U | Y |  |  | | ABS | Include |
| 100 | Mitra S (2016) | 2016 | 27367608 | Case report | Y | U | Y | Y | Y | U | U | Y |  |  | | ABS | Include |
| 101 | Barroca H (2007) | 2007 | 17536559 | Case report | Y | Y | Y | Y | Y | Y | U | Y |  |  | | ABS | Include |
| 102 | Ho AS (2011) | 2011 | — | Case report | Y | U | Y | Y | U | U | U | Y |  |  | | ABS | Include with reporting limitations |
| 103 | Kim SW (2013) | 2013 | 24082228 | Case report | Y | Y | Y | Y | Y | Y | U | Y |  |  | | FT | Include |
| 104 | Sánchez-Romero C (2018) | 2018 | 29624716 | Case report | Y | U | Y | Y | Y | U | U | Y |  |  | | ABS | Include |
| 105 | Patel SN (2019) | 2019 | 31371556 | Case report | Y | U | Y | Y | Y | U | U | Y |  |  | | ABS | Include |
| 106 | Derner BS (2020) | 2020 | 32958356 | Case report | Y | Y | Y | Y | Y | Y | U | Y |  |  | | ABS | Include |
| 107 | Margariti P (2023) | 2023 | 36923389 | Case report | Y | Y | Y | Y | Y | U | U | Y |  |  | | FT | Include |
| 108 | Wang LM (2010) | 2010 | 20846504 | Case report | Y | U | Y | Y | Y | Y | U | Y |  |  | | ABS | Include |
| 109 | Moon HR (2015) | 2015 | 25996675 | Case report | Y | U | Y | Y | N | Y | U | Y |  |  | | ABS | Include |
| 110 | Al-Antary E (2022) | 2022 | 34387630 | Case report | Y | U | Y | Y | Y | Y | U | Y |  |  | | ABS | Include |
| 111 | Takeuchi M (2009) | 2009 | 20158626 | Case report | Y | U | Y | Y | Y | U | U | Y |  |  | | ABS | Include |
| 112 | Yagasaki H (2017) | 2017 | 28500427 | Case report | Y | U | Y | Y | Y | Y | U | Y |  |  | | ABS | Include |
| 113 | Mustafa OM (2020) | 2020 | 30813780 | Case report | Y | U | Y | Y | Y | Y | U | Y |  |  | | ABS | Include |
| 114 | Kaisho J (2020) | 2020 | 32540832 | Case report | Y | Y | Y | Y | U | Y | U | Y |  |  | | ABS | Include |
| 115 | Castro-Gago M (2009) | 2009 | 19135628 | Case report | Y | U | Y | Y | Y | U | U | Y |  |  | | ABS | Include |
| 116 | Eissa SS (2020) | 2020 | 32609843 | Case report | Y | Y | Y | Y | Y | Y | U | Y |  |  | | FT | Include |
| 117 | de Soccio G (2020) | 2020 | 32505931 | Case report | Y | Y | Y | Y | U | U | U | Y |  |  | | ABS | Include with reporting limitations |
| 118 | Hughes DB (2006) | 2006 | 16445413 | Case report | Y | U | Y | Y | Y | Y | U | Y |  |  | | ABS | Include |
| 119 | Nishi T (2007) | 2007 | 17660992 | Case report | Y | U | Y | Y | Y | U | U | Y |  |  | | ABS | Include |
| 120 | Dincaslan HU (2008) | 2008 | 18668676 | Case report | Y | U | Y | Y | Y | U | U | Y |  |  | | ABS | Include |
| 121 | Kanoff JM (2010) | 2010 | 20458239 | Case report | Y | U | Y | Y | Y | U | U | Y |  |  | | ABS | Include |
| 122 | Prakash G (2012) | 2012 | 22367388 | Case report | Y | U | Y | Y | Y | U | U | Y |  |  | | ABS | Include |
| 123 | Jain D (2013) | 2013 | 23025555 | Case report | Y | U | Y | Y | Y | U | U | Y |  |  | | ABS | Include |
| 124 | Heintz D (2015) | 2015 | 25625580 | Case report | Y | U | Y | Y | Y | U | U | Y |  |  | | FT | Include with reporting limitations |
| 125 | Kiratli H (2016) | 2016 | 26874167 | Case report | Y | U | Y | Y | U | Y | U | Y |  |  | | ABS | Include with reporting limitations |
| 126 | Chakraborty R (2017) | 2017 | 28512266 | Case report | Y | U | Y | Y | Y | Y | U | Y |  |  | | ABS | Include |
| 127 | Torres Pérez P (2018) | 2018 | 29797066 | Case report | Y | U | Y | Y | Y | U | U | Y |  |  | | ABS | Include with reporting limitations |
| 128 | Rouatbi A (2019) | 2019 | 31221451 | Case report | Y | Y | Y | Y | Y | Y | U | Y |  |  | | REC | Include |
| 129 | Yong GY (2020) | 2020 | 32511962 | Case report | Y | U | Y | Y | Y | U | U | Y |  |  | | ABS | Include with reporting limitations |
| 130 | Mifsud M (2021) | 2021 | 34245213 | Case report | Y | Y | Y | Y | Y | Y | U | Y |  |  | | ABS | Include |
| 131 | Verma C (2022) | 2022 | 35695144 | Case report | Y | U | Y | Y | Y | Y | U | Y |  |  | | ABS | Include |
| 132 | Kumar P (2023) | 2023 | 36861607 | Case report | Y | U | Y | Y | Y | Y | U | Y |  |  | | ABS | Include |
| 133 | Yaguchi A (2024) | 2024 | 38605542 | Case report | Y | U | Y | Y | Y | Y | U | Y |  |  | | ABS | Include |
| 134 | Meyer P (2018) | 2018 | 29468209 | Case report | Y | Y | Y | Y | Y | Y | U | Y |  |  | | FT | Include |
| 135 | Unresolved #1 | — | — | Unresolved primary report | U | U | U | U | U | U | U | U |  |  | | — | Seek further information |
| 136 | Unresolved #2 | — | — | Unresolved primary report | U | U | U | U | U | U | U | U |  |  | | — | Seek further information |
| 137 | Unresolved #3 | — | — | Unresolved primary report | U | U | U | U | U | U | U | U |  |  | | — | Seek further information |
| 138 | Unresolved #4 | — | — | Unresolved primary report | U | U | U | U | U | U | U | U |  |  | | — | Seek further information |

Item summary, case reports (n = 122): Q1: Y 122, N 0, U 0, NA 0 | Q2: Y 73, N 0, U 49, NA 0 | Q3: Y 122, N 0, U 0, NA 0 | Q4: Y 122, N 0, U 0, NA 0 | Q5: Y 110, N 1, U 11, NA 0 | Q6: Y 86, N 0, U 36, NA 0 | Q7: Y 11, N 0, U 111, NA 0 | Q8: Y 122, N 0, U 0, NA 0

Item summary, abstract/poster-level reports (n = 3, eight-item checklist, abstract-limited): Q1: Y 2, N 0, U 1, NA 0 | Q2: Y 1, N 0, U 2, NA 0 | Q3: Y 2, N 0, U 1, NA 0 | Q4: Y 2, N 0, U 1, NA 0 | Q5: Y 2, N 0, U 1, NA 0 | Q6: Y 2, N 0, U 1, NA 0 | Q7: Y 0, N 0, U 3, NA 0 | Q8: Y 2, N 0, U 1, NA 0

Item summary, case series (n = 7): Q1: Y 4, N 0, U 3, NA 0 | Q2: Y 7, N 0, U 0, NA 0 | Q3: Y 7, N 0, U 0, NA 0 | Q4: Y 0, N 0, U 7, NA 0 | Q5: Y 0, N 0, U 7, NA 0 | Q6: Y 7, N 0, U 0, NA 0 | Q7: Y 7, N 0, U 0, NA 0 | Q8: Y 5, N 0, U 2, NA 0 | Q9: Y 7, N 0, U 0, NA 0 | Q10: Y 0, N 7, U 0, NA 0

Checklist items — Case Report: Q1, demographic characteristics clearly described; Q2, patient history clearly described; Q3, current clinical condition clearly described; Q4, diagnostic tests or assessment methods and results clearly described; Q5, interventions or treatment procedures clearly described; Q6, post-intervention clinical condition clearly described; Q7, adverse events or unanticipated events identified and described; Q8, takeaway lessons clearly described. Case Series: Q1, clear criteria for inclusion; Q2, condition measured in a standard, reliable way; Q3, valid identification of the condition; Q4, consecutive inclusion; Q5, complete inclusion; Q6, demographics clearly reported; Q7, clinical information clearly reported; Q8, outcomes or follow-up clearly reported; Q9, demographic information of presenting sites clearly reported; Q10, statistical analysis appropriate.

Q10 (statistical analysis) was coded Not applicable for all seven case series because none included statistical analysis.

The four unresolved and two pending-identification entries are retained transparently: their unique primary-report mapping could not be confidently resolved, so no unsupported JBI domain ratings were assigned. This unresolved mapping does not create additional patients beyond N = 147.

Where no author is shown, the study is identified by its title (author not available in the accessible record).

Year or PMID shown as ‘—’: not available in the accessible record.

Overall appraisal classes: Include; Include with reporting limitations; Seek further information; Not appraisable. Source: FT, full text; ABS, abstract, poster archive, or bibliographic record; —, primary report not yet uniquely identified.

Items with study design ‘To verify’ or ‘Unresolved primary report’ (n = 6) are presented transparently and are not included in the item summaries. The 150 included reports were mapped to 144 database records; after merging six multi-database versions of the same publication, 138 distinct primary-report units remained (see Units of analysis).

Units of analysis: the table lists 138 primary-report units. Multi-database records of the same publication were merged (multi-database versions for Bilgin 2021, Kahn 2020, Ferguson 2015, Calderon 2021, Nakasu 2007, and Masui 2012), so report-level units differ from the number of database records retrieved. The four unresolved entries correspond to supplementary screening patients whose primary reports await identification; they are retained transparently and do not affect the primary malignancy-mimic analysis, which was based on verified records. One case report (E0866, PMID 25996675) received a Q5 rating of No because no therapeutic intervention was documented in the accessible record.
